# Current status and trends in the study of intestinal flora in cognitive disorders: a bibliometric and visual analysis

**DOI:** 10.3389/fmicb.2025.1577597

**Published:** 2025-05-27

**Authors:** Qi Zhang, Zhenmei Gao, Yunqing Deng, Xiangqing Xu, Wenyu Sun, Rui Liu, Tianao Zhang, Xilei Sun

**Affiliations:** ^1^College of Rehabilitation Medicine, Shandong University of Traditional Chinese Medicine, Jinan, Shandong, China; ^2^Department of Rehabilitation Physiotherapy, Affiliated Hospital of Shandong University of Traditional Chinese Medicine, Jinan, Shandong, China; ^3^College of Special Education and Rehabilitation, Binzhou Medical College, Yantai, Shandong, China; ^4^Affiliated Hospital of Shandong University of Traditional Chinese Medicine, Jinan, Shandong, China

**Keywords:** intestinal flora, cognitive impairment, gut-brain axis, short-chain fatty acids, fecal microbiota transplantation techniques, bibliometrics

## Abstract

**Background:**

Cognitive impairment is a decline in people’s ability to think, learn, and remember, which has some impact on an individual’s daily activities or social functioning. Microbial toxins and metabolites from dysregulated gut microbiota directly interact with the intestinal epithelium. This interaction triggers neuroinflammation and neurodegeneration in the central nervous system, ultimately impairing cognitive function. It has been found that modulation of gut flora can be an effective intervention to improve cognitive dysfunction. This study is the first to summarize and outline the global research status and trends in this field from a bibliometric perspective, providing reference and guidance for future research in this field.

**Methods:**

Based on the Web of Science Core Collection (WoSCC) database, Literature on gut flora and cognitive impairment published between 1999–2025 was searched. Bibliometric analysis was performed using VOSviewer and CiteSpace software to analyze the data on countries, institutions, authors, journals, keywords, citations, and to generate visual maps.

**Results:**

A total of 1,702 pieces of related literature were retrieved. The overall trend of publication is increasing. China has published the largest number of papers, and Huazhong University of Science & Technology and Kim, Dong-Hyun were the institutions and individuals with more publications. The most frequently cited journal is *SCI REP-UK*. The most frequent keywords are gut microbiota, followed by Alzheimer’s disease, cognitive impairment, Brain, oxidative stress and Inflammation.

**Conclusion:**

In recent years, the research application of gut flora in the treatment of cognitive impairment has made remarkable progress. Oxidative stress and inflammatory response have become the main research hotspots for gut flora to improve cognitive impairment in patients. The gut-brain axis plays an important role in the study of the mechanism of action. Short-chain fatty acids are the focus of research on gut microbial metabolism. Fecal microbial transplantation technology is increasingly being used as an emerging method for the application of intestinal flora. Modifying the gut flora by modifying diet and exercise may be an effective strategy to prevent and improve cognitive dysfunction in the future. Future studies may focus more on gender differences in the role of gut flora in the modulation of cognitive function.

## 1 Introduction

Cognitive impairment is characterized by the aging process in which the frontal and temporal lobes of the brain atrophy, leading to a decrease in synaptic density and remodeling of neural circuits, which in turn leads to cognitive decline, manifested as a decrease in an individual’s ability to think, learn, and remember (Blinkouskaya and Weickenmeier, 2021). Even severe cases of dementia (Wang et al., 2024). The global prevalence of Mild Cognitive Impairment (MCI) is 19.7% and is increasing over time, rising significantly to 32.1% after 2019 (Song et al., 2023). With the increasing aging of the population, cognitive impairment has become an important challenge in global public health and there is an urgent need to explore innovative intervention strategies. The causes of cognitive impairment include oxidative stress, mitochondrial dysfunction, and neuroinflammatory factors (Sorrentino et al., 2017), the molecular pathologies include: (1) oxidative stress-induced neuronal DNA damage, with abnormally elevated levels of the characteristic biomarker, 8-hydroxydeoxyguanosine (Liu et al., 2017); (2) reduced activity of mitochondrial complex IV, which triggers insufficient production of ATP (Kriebel et al., 2020); (3) Microglia overactivation drives the release of TNF-*α*, IL-1β and other pro-inflammatory factors in a cascade, inducing a neuroinflammatory cascade (Jung et al., 2023).

With the innovation of microbiological research techniques, the association between gut flora and cognitive impairment has become a hot research topic. The gastrointestinal tract contains trillions of commensal microorganisms belonging to more than 1,000 species, which coexist harmoniously with the host, participate in the digestion and absorption of nutrients (Guo et al., 2022)and regulate metabolic functions through molecular exchanges with the host (Ji et al., 2015). In recent years, intestinal flora has been recognized as a “second brain.” It interacts with the host through metabolites and immunomodulatory molecules, forming a complex bidirectional communication network known as the gut-brain axis (Cryan et al., 2020). Gut flora not only participates in the synthesis of neurotransmitters, hormones and other bioactive molecules and a variety of metabolic processes, but also plays a key role in maintaining the integrity of the intestinal barrier and blood–brain barrier (Rowland et al., 2018). Studies indicate that gut microbiota dysbiosis may contribute to neurodegenerative disorders (Bicknell et al., 2023), and potential pathways affecting cognitive function include: (1) pro-inflammatory factors and reactive oxygen species (ROS) released by aberrant flora penetrate the blood–brain barrier, inducing microglial cell activation and neuronal damage (Chen et al., 2021); (2) short-chain fatty acids (SCFAs) are known to affect cognitive function through the regulation of histone de-acetylase (HDAC). And SCFAs affect synaptic plasticity and expression of neurotrophic factors by regulating histone deacetylase activity (Silva et al., 2020); (3) Microbial toxins and metabolites produced by abnormal bacterial flora act on the intestinal epithelium, leading to an increase in the permeability of the intestinal barrier (Antushevich, 2020), which then destroys the function of the blood–brain barrier, and induces neuroinflammation and neurodegeneration in the central nervous system (Liang et al., 2018).

Bibliometric analysis of the field can visualize the development trend and dynamics of a certain field, reflecting the current research hotspots and cutting-edge directions (Chen and Song, 2019). Although the number of studies on intestinal flora and cognitive impairment has increased rapidly in the past two years, there is still a lack of studies revealing the hotspots and trends in this field based on visualization analysis. Based on the Web of Science Core Collection database, this study used CiteSpace software to analyze the relevant literature and draw visual maps, aiming to reveal the development history, research hotspots and future trends in this field.

## 2 Methodology

### 2.1 Data sources

**Figure 1:**
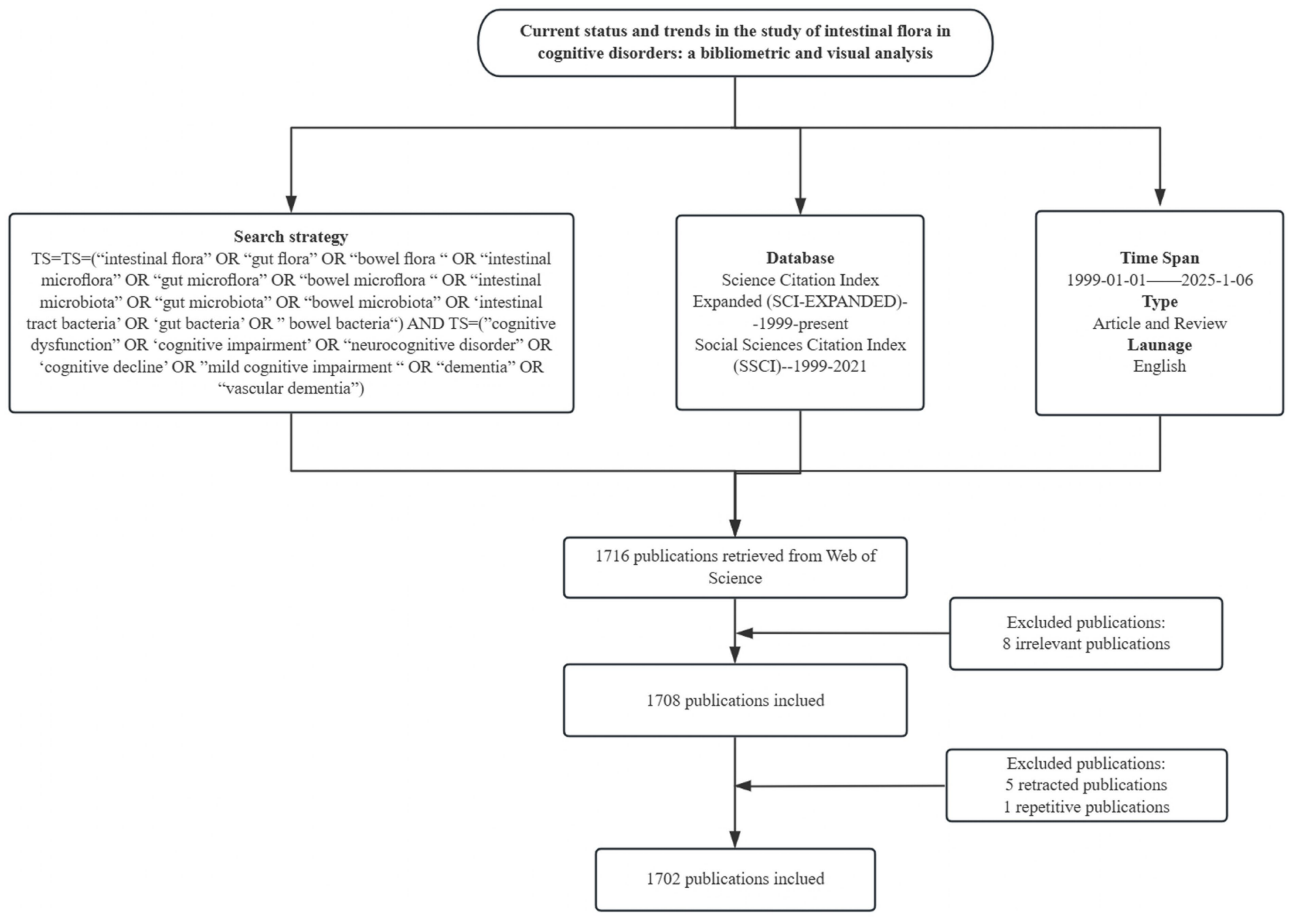
Data retrieval and analysis process.

This study was based on Web of Science core ensemble database, literature screening from Science Citation Index Expanded (SCI-EXPANDED)--1999-present, Social Sciences Citation Index (SSCI)---1999–2021 two core databases, the search terms were TS = (“intestinal flora” OR “gut flora” OR “bowel flora “OR “intestinal microflora” OR “gut microflora” OR “bowel microflora “OR “intestinal microbiota” OR “gut microbiota” OR “bowel microbiota” OR ‘intestinal tract bacteria’ OR ‘gut bacteria’ OR” bowel bacteria“) AND TS = (“cognitive dysfunction” OR ‘cognitive impairment’ OR “neurocognitive disorder” OR ‘cognitive decline’ OR” mild cognitive impairment “OR “dementia” OR “vascular dementia”), The search period was set from January 1999 to January 6, 2025, and the language was set to English. Although the search period was set from January 1999 to January 2025, the first relevant literature in the field was screened and found to be published in 2012, so the actual time span for the inclusion of literature was from 2012 to 2025. Initially, 1716 articles were searched in the literature, and the article types were limited to Article and Review. By reading the titles and abstracts of the articles, 8 articles that were not related to the research topic, 1 duplicate article, and 5 retracted articles were removed. After the above screening process, 1702 documents were finally included, from which data such as country, institution, author, journal, keywords, and citation information were extracted for bibliometric analysis. As shown in Figure 1.

### 2.2 Inclusion and exclusion criteria

*Inclusion criteria*: (1) The study should also address the association between “gut flora” (such as gut microbiota, flora metabolites, etc.) and “cognitive disorders” (such as Alzheimer’s disease, mild cognitive impairment, etc.), and exclude literature discussing only a single topic. Literature; (2) Language limited to English to ensure the analyzability and consistency of the data; (3) Timeframe from January 1999 to January 2025, covering the field from the early stage to the latest progress of the research.

*Exclusion criteria*: (1) non-research-based literature (such as editorials, conference abstracts, reviews, news, etc.); (2) literature not in English or full text unavailable (3) duplicated publications or overlapping data; (4) withdrawn or academically controversial literature; and (5) literature with research content unrelated to the topic.

### 2.3 Research tools

This study mainly used VOSviewer and CiteSpace software for bibliometric analysis. VOSviewer is a visual analysis tool introduced by Van Eck and Waltman from Leiden University, The Netherlands in 2009 (van Eck and Waltman, 2017). VOSviewer (V1.6.19) was used to perform visual analysis of the country, institution, author, keyword, literature co-citation, and journal co-citation. Parameters configured in VOSviewer were adjusted according to the actual situation. CiteSpace was created in 2004 by Dr. Chiu-Mei Chen and his team at Drexel University in Philadelphia, USA, using a time-based and graphical visualization visualization and analysis tool. It can present the development trend and structural relationship of scientific knowledge, visualize the development trend and dynamics of a certain field, and reflect the current research hotspots and cutting-edge directions (Chen and Song, 2019). Keyword clustering analysis, keyword timeline graphs, and journal biplot overlays were performed using CiteSpace (V.6.4.R1). The parameters of the CiteSpace software were set as follows: time slices (1 year), *K* = 25, network pruning method was the Pathfinder method, and keyword clustering was performed using the LLR clustering algorithm.

## 3 Results

### 3.1 Number of annual publications

**Figure 2:**
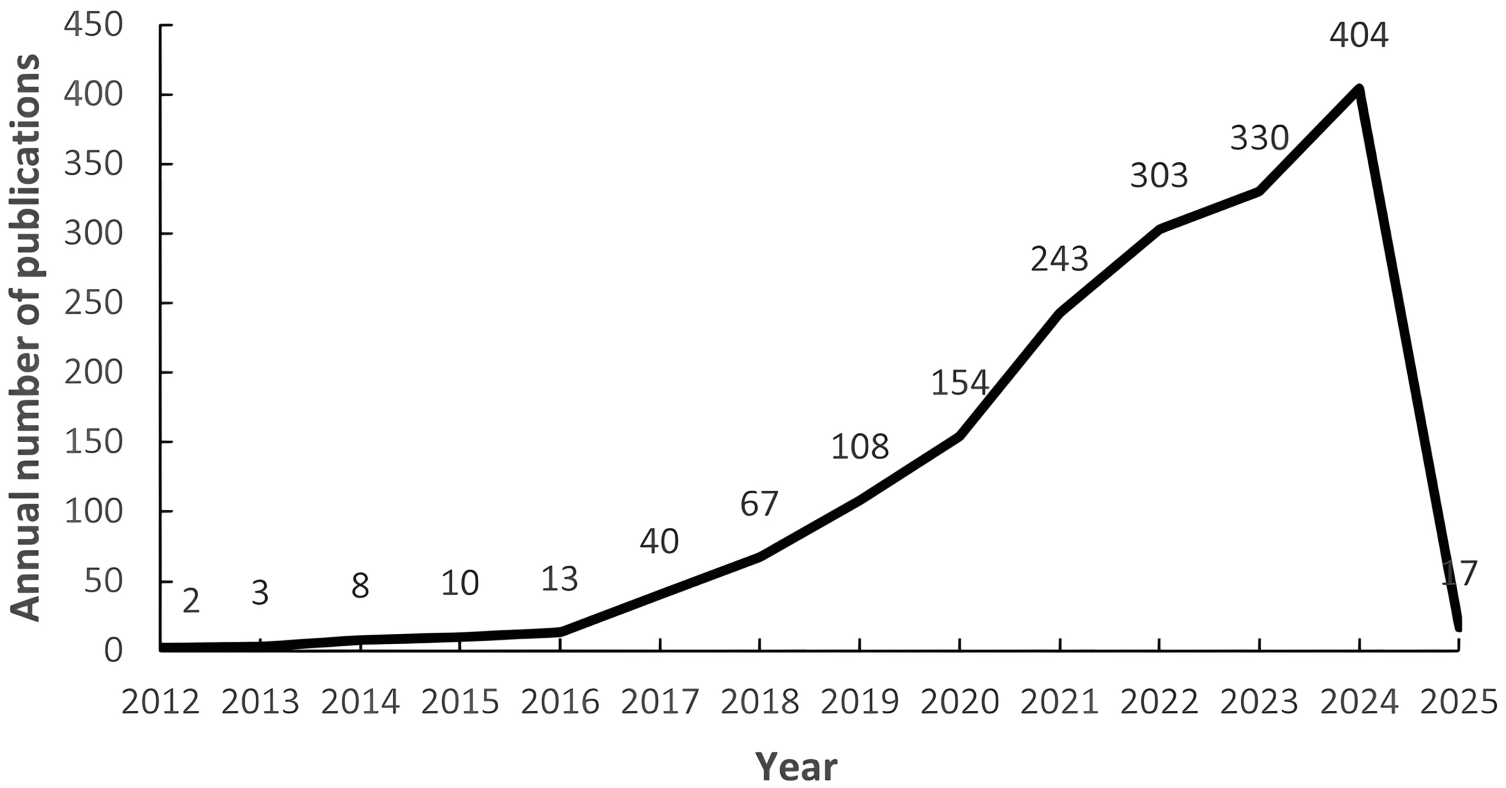
Number of annual publications from 1999 to 2025. The numbers on the left represent the number of articles issued per year, and the numbers below represent the year.

After screening, a total of 1,702 related papers were included. Figure 2 shows the annual publication volume and the trend of change, and the field shows a remarkable three-stage development: (1) the budding period (2012–2016): the annual publication volume is relatively small, and the first core literature was published in 2012; (2) the development period (2017–2020): the annual publication volume is gradually increasing, and the volume of publications exceeded 150 in 2020; (3) the explosion period (2021–2024): the annual publication volume jumped from 243 (in 2021) to 404 (in 2024), the number of annual articles jumps from 243 (in 2021) to 404 (in 2024). The growth rate accelerates significantly from 2021 onwards, which indicates that the research heat of intestinal flora and cognitive impairment has increased significantly in the past decade and has become an important research direction in this field.

### 3.2 Countries/regions

**Figure 3:**
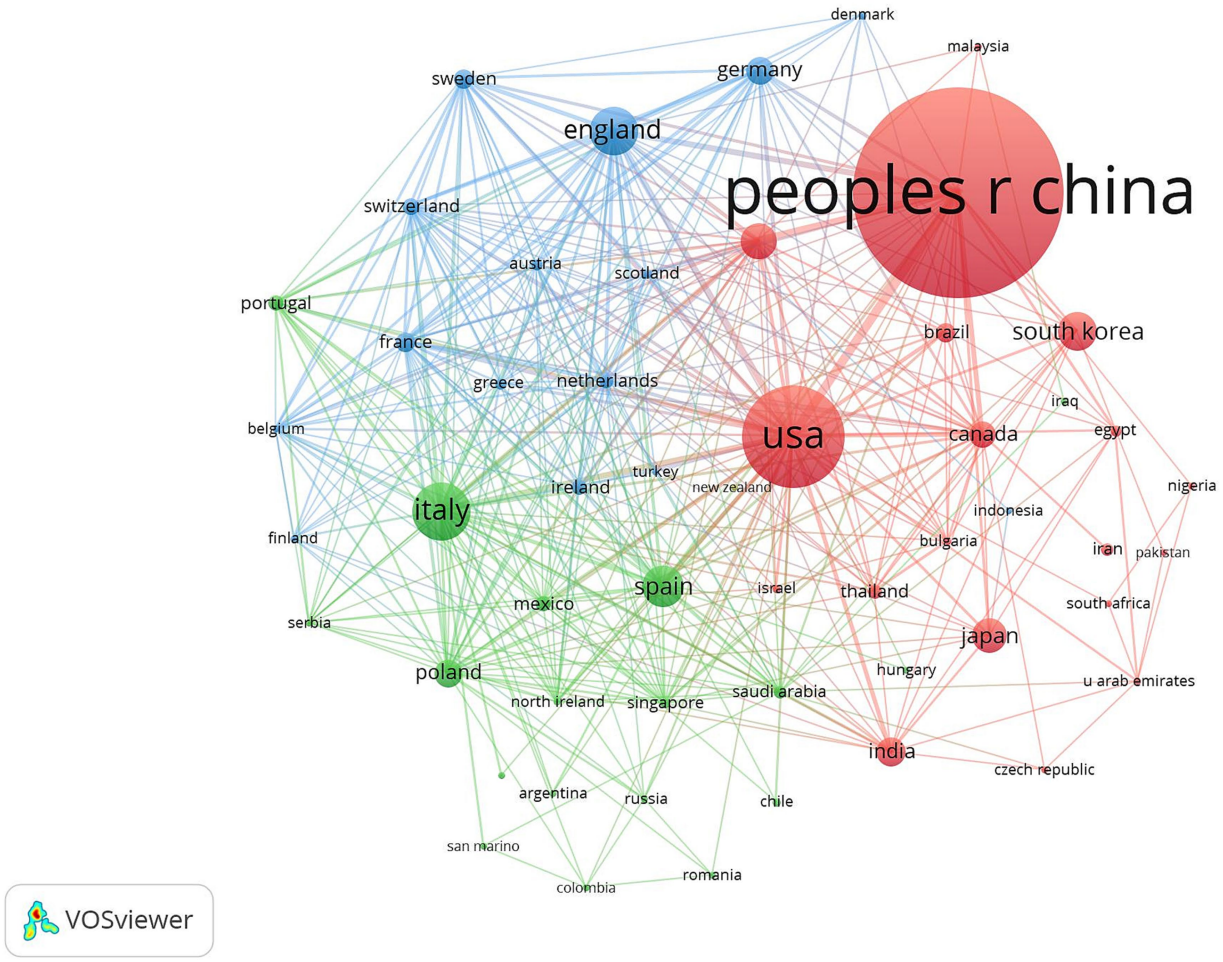
Cluster-based countries/regions collaboration map. The diameter of the circle is positively correlated with the number of national/regional publications, while the density of the connecting lines visualizes the intensity of national/regional cooperation.

**Figure 4:**
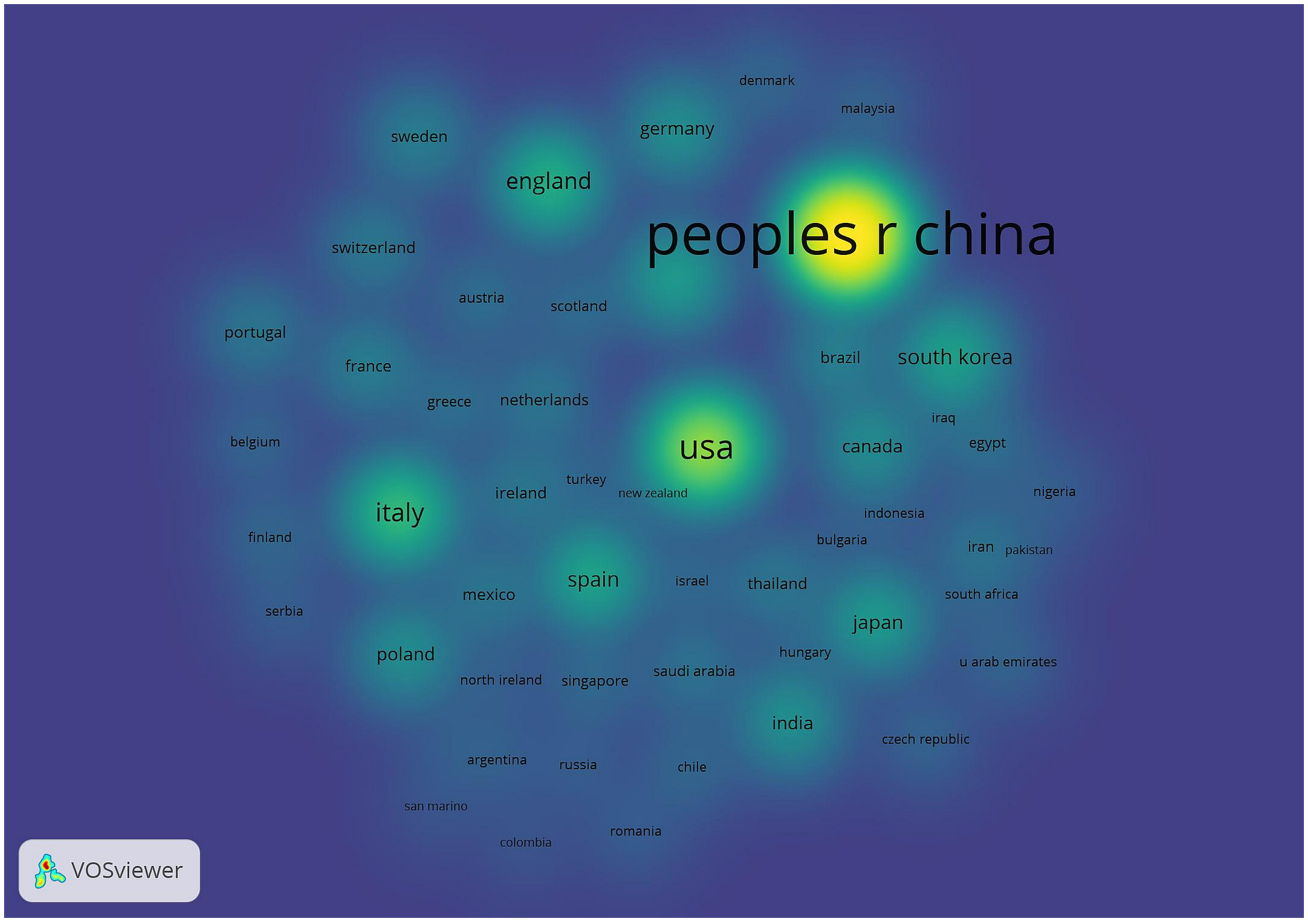
Countries/regions density map. The node size is related to the number of articles published in the country/region, and the node color is related to the number of articles published in recent years, the more the number of articles, the higher and the color brightness.

Table 1 shows the top five countries/regions in terms of number of publications and centrality. The analysis shows that China ranks first with 798 publications, while the United States (287) and Italy (132) rank second and third, respectively. In terms of the centrality index, Australia (0.81), Saudi Arabia (0.71) and the Netherlands (0.47) are in the top three, showing high network centrality. Although China has laid the foundation for research in this field by virtue of its large number of publications, but its centrality does not rank among the top five; the United States excels in both publication volume and centrality, reflecting the balanced advantages of academic output scale and academic cooperation; Australia’s high centrality indicates that it plays a key role as a pivotal hub in the field of the discipline, effectively connecting the research clusters in North America, Europe, and Asia-Pacific, and playing an important role in promoting cross-regional academic cooperation and dissemination of theories. The high centrality of Australia suggests that it plays a key role as a hub in the discipline, effectively connecting research clusters in North America, Europe and the Asia-Pacific region, and playing an important role in promoting cross-regional academic cooperation and theory dissemination. Figure 3 shows that the country/region collaboration network maps visualize the global research cooperation pattern, which shows that there is a close collaboration network among countries/regions. Figure 4 shows that China has a high research intensity in this field, and its research investment and achievements are remarkable.

**Table 1:** Top 5 countries/regions in terms of the number of publications.

Ranking	Publications	Country/Region	Centrality	Country/Region
1	798	PEOPLES R CHINA	0.81	AUSTRALIA
2	287	USA	0.71	SAUDI ARABIA
3	132	ITALY	0.47	NETHERLANDS
4	94	ENGLAND	0.38	BELGIUM
5	84	SPAIN	0.36	SCOTLAND

### 3.3 Institutions

**Figure 5:**
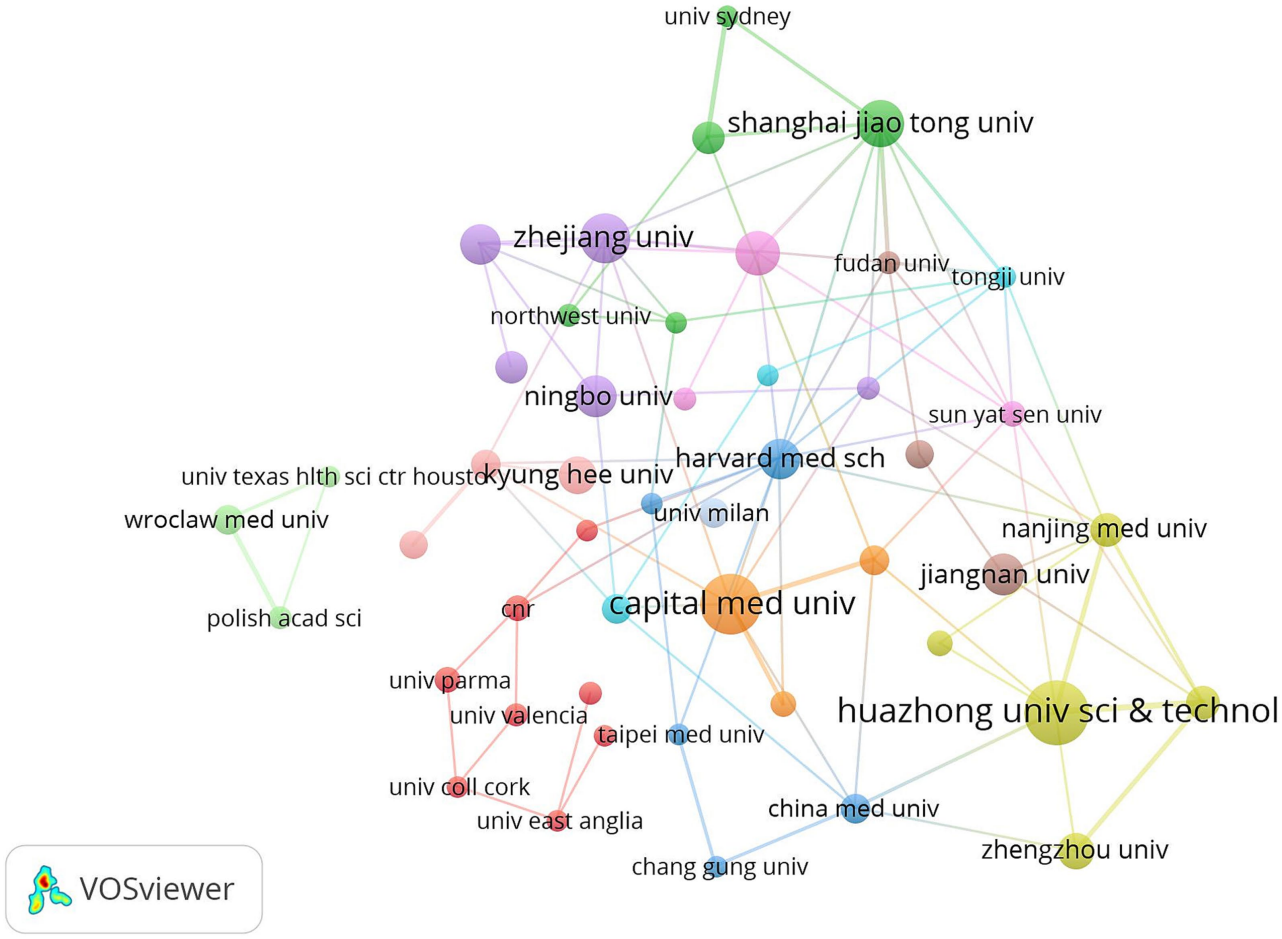
Cluster-based institutional collaboration map. The diameter of the circle is positively correlated with the number of papers issued by the institution, while the density of the connecting lines visualizes the intensity of institutional cooperation.

**Figure 6:**
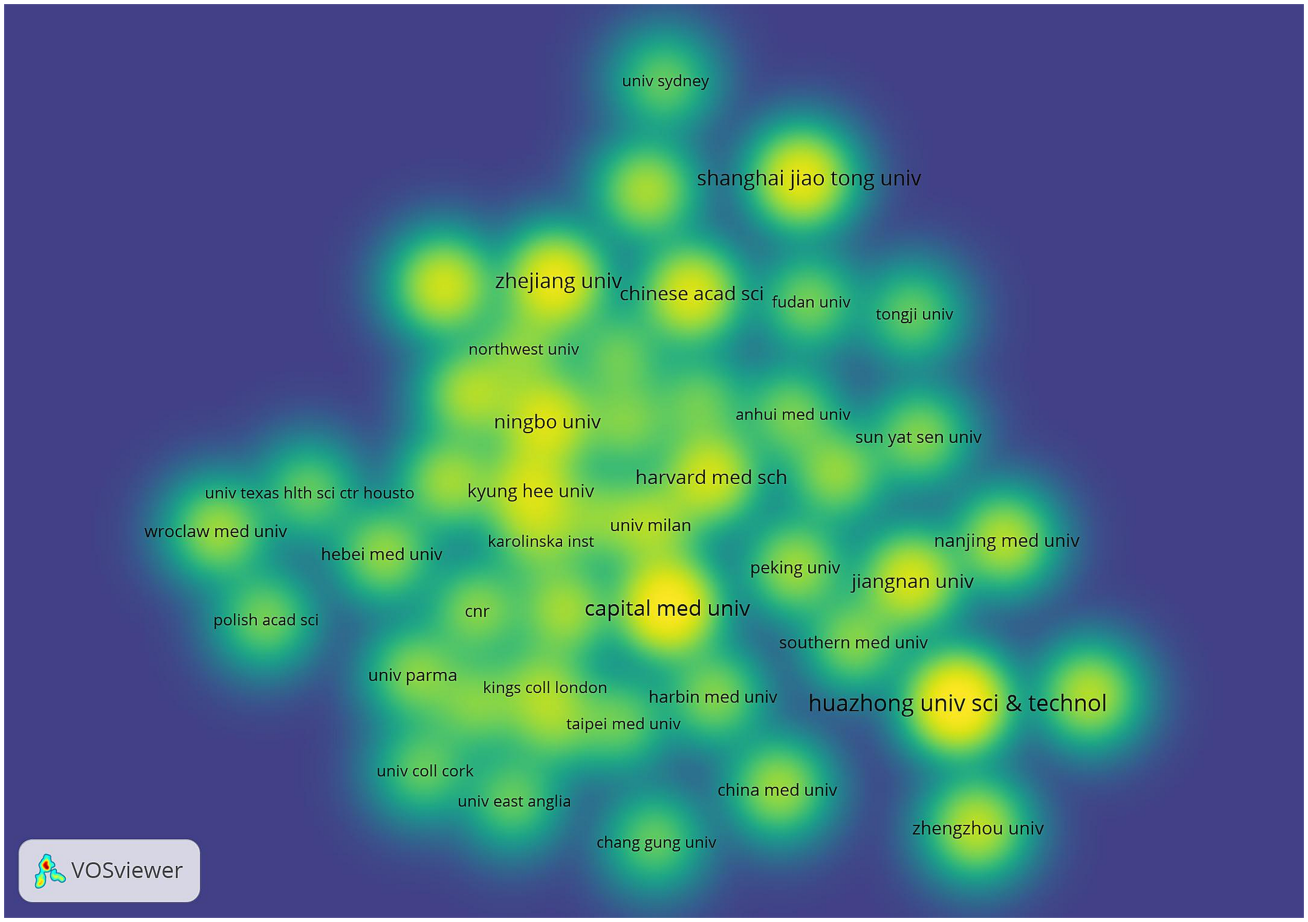
Institutional density map. The node size is related to the number of publications of the institution, and the node color is related to the number of publications in recent years, the more publications the higher the color brightness.

As can be seen from Figure 5 Institutional Co-occurrence Network Mapping and Figure 6 Hot Spot Mapping, Huazhong University of Science & Technology is the institution with the largest number of publications and higher influence in this field, with a total of 41 papers published. Capital Medical University and Harvard Capital Medical University and Harvard University follow closely with 38 and 33 papers respectively, and Harvard Medical School, Boston University and Zhejiang University are the top three institutions in the centrality ranking, with 0.47, 0.44 and 0.3 respectively, which indicates that the exchange and cooperation between this institution and other institutions are relatively close. Table 2 shows the top 10 institutions in terms of the number of articles and centrality. In the hotspot map of institutions, Huazhong University of Science & Technology pays more attention to the field. Although Chinese institutions have an advantage in the number of articles (41 articles from Huazhong University of Science & Technology and 27 articles from Zhejiang University), their institutional centrality is low, and this phenomenon of “high output-low centrality” reflects that the current research in the field of intestinal flora and cognitive function in China is facing a serious challenge in the globalized knowledge network.

**Table 2:** Top 10 institutions in terms of the number of publications.

Ranking	Publications	Institution	Centrality	Institution
1	41	Huazhong University of Science & Technology	0.47	Harvard Medical School
2	38	Capital Medical University	0.44	Boston University
3	33	Harvard University	0.3	Zhejiang University
4	31	Chinese Academy of Sciences	0.3	Brigham & Women’s Hospital
5	30	University of California System	0.22	Columbia University
6	30	University of London	0.22	Collaborative Innovation Center for Diagnosis & Treatment of Infectious Diseases
7	29	University of Texas System	0.22	Institute of Microbiology
8	28	Zhejiang University	0.21	University of London
9	27	Shanghai Jiao Tong University	0.21	Jining Medical University
10	24	Veterans Health Administration (VHA)	0.2	Duke University

### 3.4 Authors

**Figure 7:**
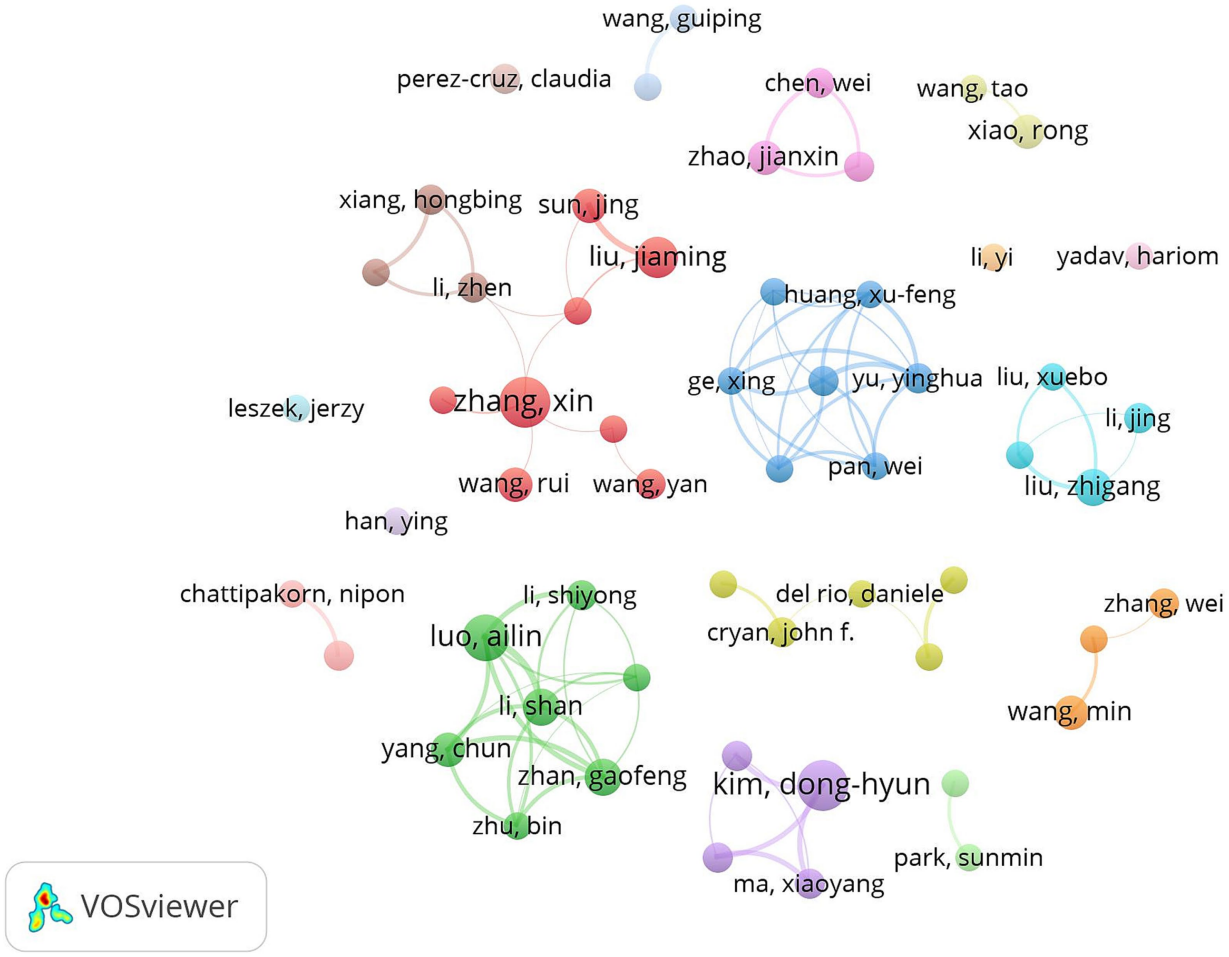
Cluster-based author collaboration map. The diameter of the circle is positively correlated with the number of publications by the authors, while the density of the connecting lines intuitively reflects the intensity of the collaboration between the authors.

**Figure 8:**
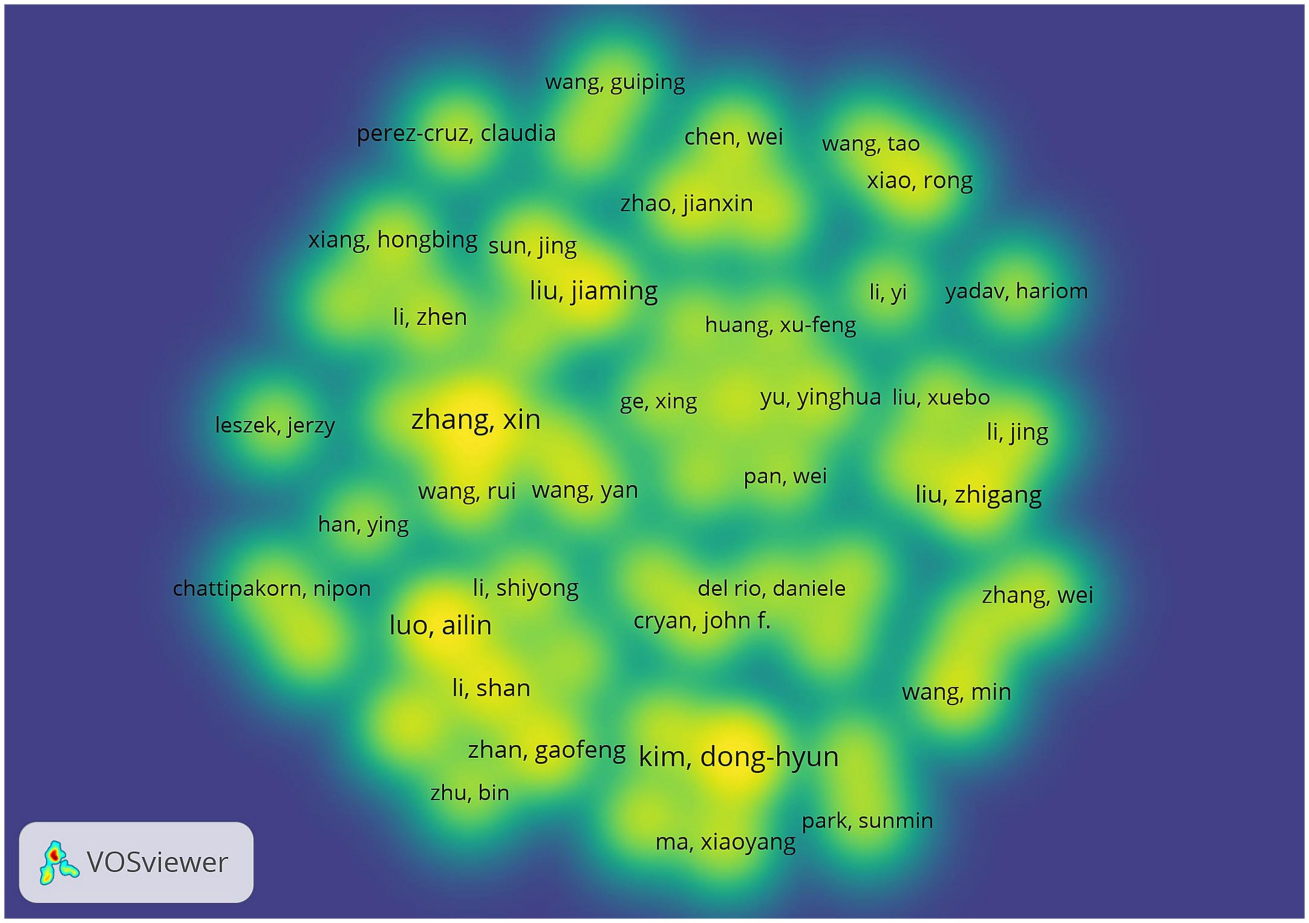
Author density map. The node size is related to the number of posts by the author, and the node color is related to the number of posts in recent years, the more posts the higher the color brightness.

Table 3 shows the details of the top 10 authors in terms of publications. Tied for the first place are Kim, Dong-Hyun, Zhang, Xin and Liu, Jiaming with 12 publications. The 4th to 10th ranks show a clear echelon distribution: Xiang, Hongbing ranked the 4th with 10 publications, followed by Yang, Chun with 8 publications, demonstrating the continuous and stable research output ability. Figures 7, 8 show that the author-based research team has formed a close cooperation network. Through resource sharing and complementary advantages, scholars have formed a close relationship with each other, and the teamwork has effectively promoted the rapid development of the field.

**Table 3:** Top 10 authors in terms of the number of publications.

Ranking	Publications	Author
1	12	Kim, Dong-Hyun
2	12	Zhang, Xin
3	12	Liu, Jiaming
4	10	Xiang, Hongbing
5	8	Yang, Chun
6	8	Li, Shan
7	8	Huang, Xu-Feng
8	7	Liu, Zhigang
9	7	Li, Zhen
10	7	Sun, Tianning

### 3.5 Keyword analysis

#### 3.5.1 Keyword co-occurrence analysis

**Figure 9:**
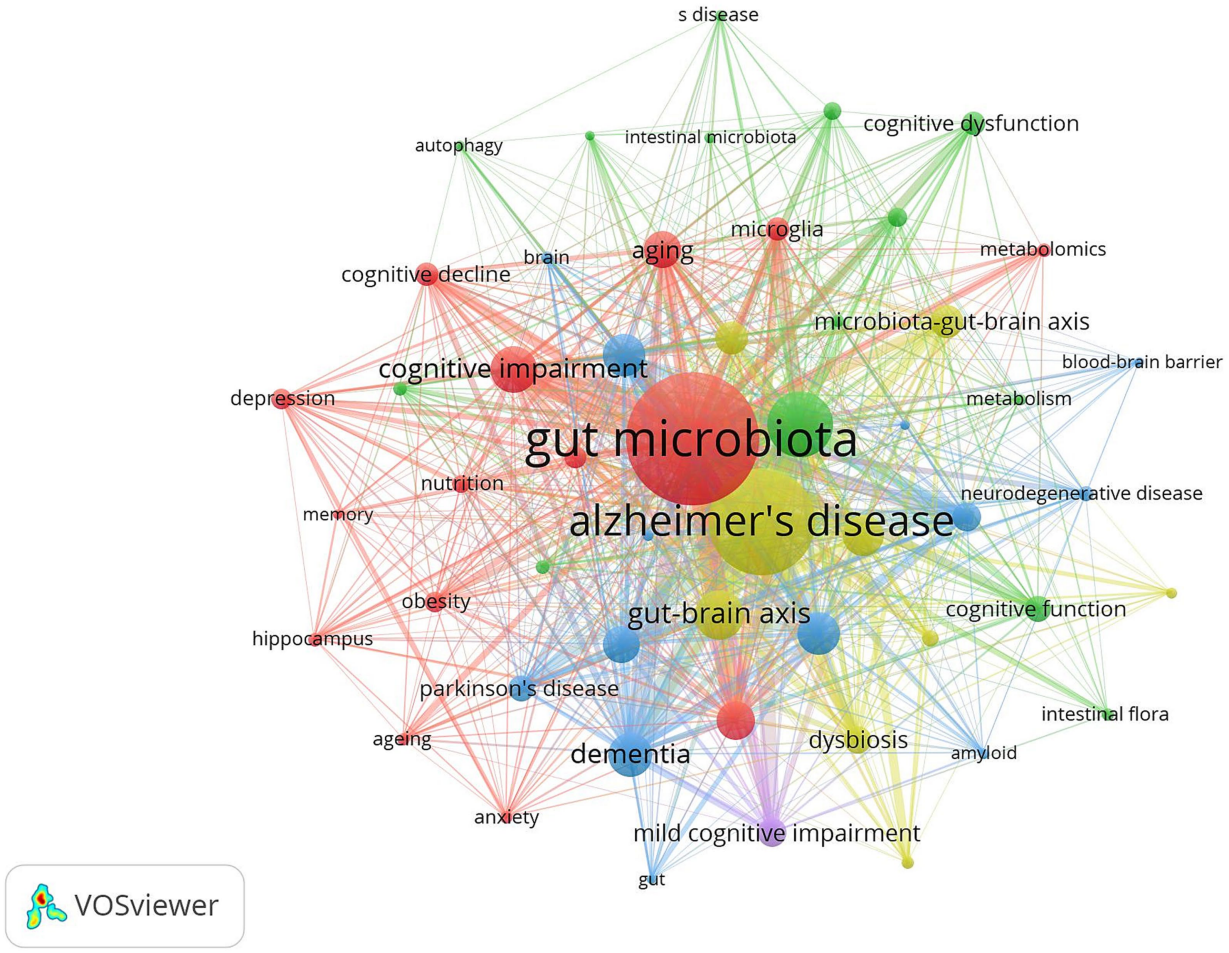
Cluster-based keywords collaboration map. The diameter of the circle is positively correlated with the frequency of the keywords, while the density of the connecting lines intuitively reflects the relevance of the keywords to each other.

Keyword co-occurrence analysis can reveal the research hotspots and trends in the field intuitively and comprehensively. The keyword co-occurrence mapping analysis in Figure 9 in terms of frequency of occurrence “gut microbiota” appears most frequently “Alzheimer’s disease” “cognitive impairment” “Brain” “oxidative stress” and “Inflammation” followed. From the analysis of centrality index “amyloid beta” has the highest centrality followed by “Behavior” and “Activation.” Table 4 lists the top 10 keywords in terms of frequency and centrality. Studies have shown that Alzheimer’s disease is a major research target in the field of gut flora and cognitive impairment which may be closely related to the climbing incidence of Alzheimer’s disease (AD) worldwide in recent years. Oxidative stress and inflammatory response as key pathological mechanisms have become hotspots for mechanistic studies; the brain-gut axis plays an important regulatory role in maintaining gut flora homeostasis and cognitive function; and short-chain fatty acids are at the core of the study of metabolites in gut flora.

**Table 4:** Top 10 keywords of word frequency.

Ranking	Counts	Keywords	Centrality	Keywords
1	976	gut microbiota	0.22	amyloid beta
2	597	Alzheimer’s disease	0.21	Behavior
3	362	cognitive impairment	0.19	Activation
4	241	Brain	0.15	Alzheimer’s disease
5	224	oxidative stress	0.15	Brain
6	217	Inflammation	0.14	Depression
7	186	mouse model	0.12	central nervous system
8	168	Dementia	0.12	Alzheimer disease
9	163	intestinal microbiota	0.12	Barrier
10	153	chain fatty acids	0.12	quality of life

#### 3.5.2 Keyword clustering analysis

**Figure 10:**
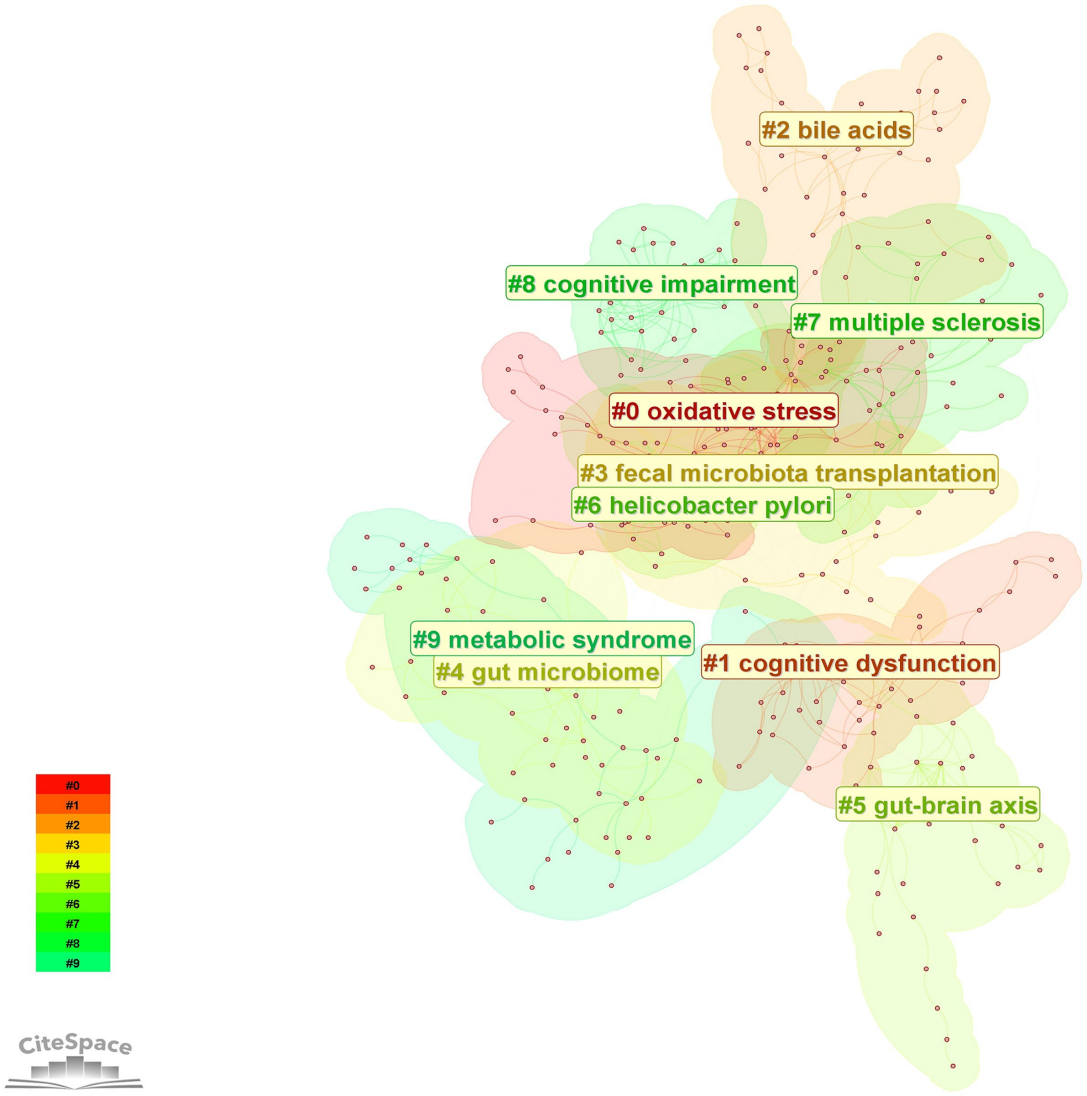
Keywords cluster map. The color blocks in the figure represent clustered categories, with different colors representing different categories.

The LLR algorithm of CiteSpace software was used to analyze the clustering of high-frequency keywords to obtain the keyword clustering diagram in Figure 10 and Table 5 presents the detailed information of the clustered keywords. The analysis results showed that the largest cluster #0 was centered on “oxidative stress” with a high contour value of 0.932, highlighting the key position of oxidative stress in the pathological mechanism. In addition, the role of bile acids in the regulation of intestinal flora is becoming more and more significant; fecal flora transplantation has become a highly regarded intervention; and the gut-brain axis, as a signaling pathway between the gut and the brain, plays an important role in the study of the mechanism of cognitive disorders affected by intestinal flora and its intervention.

**Table 5:** Keywords cluster labels and main keywords.

Cluster number	Cluster label (LLR)	Value of contour	Year	Keywords
#0	oxidative stress	0.819	2020	amyloid beta; Alzheimer disease; type 2 diabetes; metformin
#1	cognitive dysfunction	0.815	2016	intestinal microbiota; health; ps1 transgenic mice; deficits
#2	bile acids	0.941	2020	discovery; trimethylamine n-oxide; trimethylamine; vascular dementia
#3	fecal microbiota transplantation	0.844	2019	chain fatty acids; international scientific association; hypertension; glycogen synthase kinase 3 beta
#4	gut microbiome	0.764	2021	short-chain fatty acids; 16 s rrna; synbiotics; vascular cognitive impairment
#5	gut-brain axis	0.975	2018	cognition; cognitive disorder; behavior; subdiaphragmatic vagus nerve
#6	*Helicobacter pylori*	0.699	2021	model; dysfunction; sodium butyrate; cefazolin
#7	multiple sclerosis	0.863	2020	physical activity; fatty acids; artificial intelligence; mild cognitive impairment
#8	cognitive impairment	0.9	2016	gut microbiota; Alzheimer’s disease; blood–brain barrier; neurodegeneration
#9	metabolic syndrome	0.889	2020	intestinal flora; ketogenic diet; cells; disease

#### 3.5.3 Keyword timeline chart

**Figure 11:**
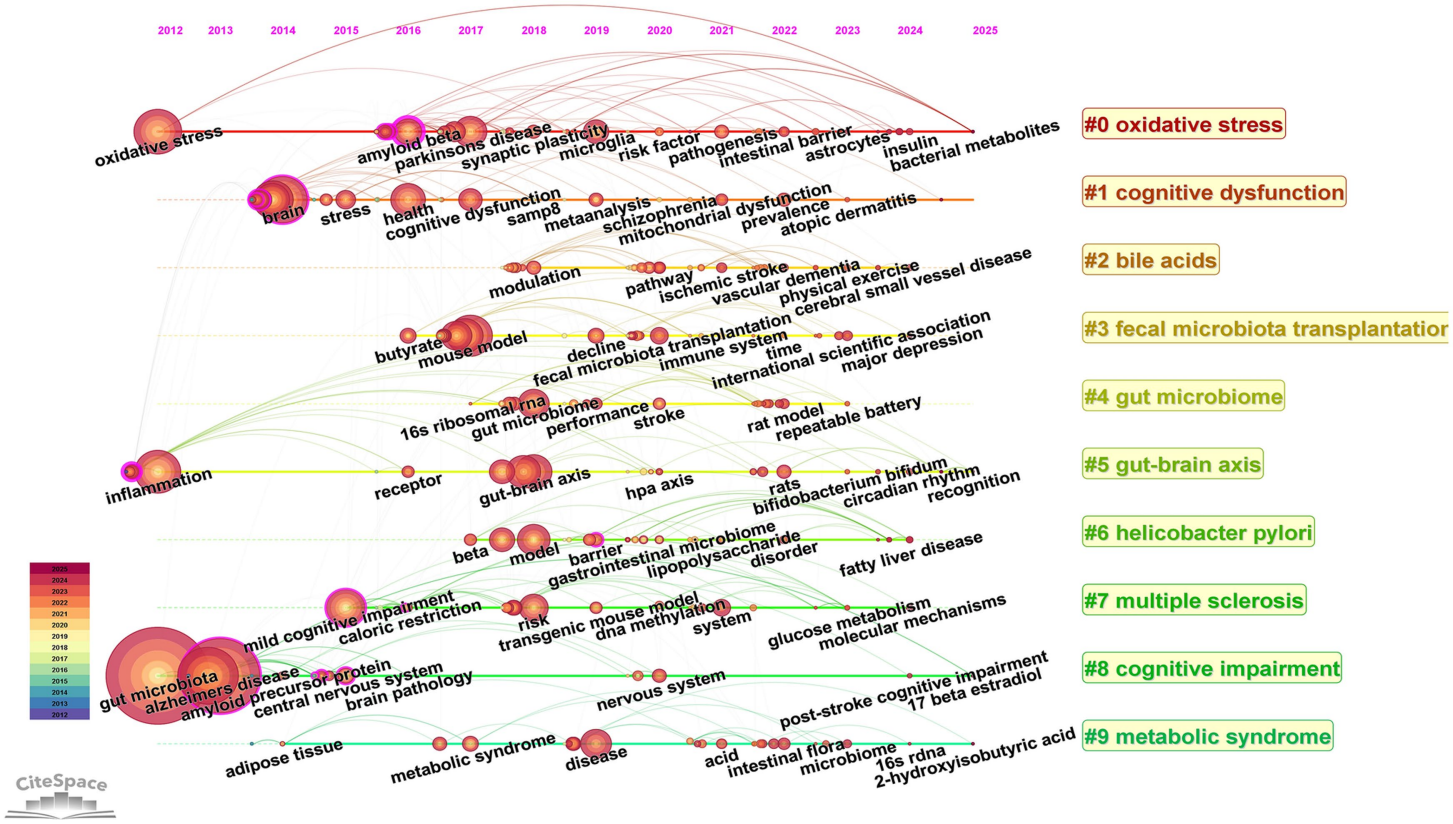
Timeline view of keywords. The horizontal axis indicates the time range, the keyword information for each cluster is indicated on the vertical axis, and the node size indicates the frequency of the keyword.

The keyword timeline diagram shows the hotspots and trends in the research field from the perspective of historical evolution. The time evolution analysis in Figure 11 reveals the dynamic evolution of research paradigms on gut flora and cognitive disorders: oxidative stress as an early research hotspot has continued to be of great interest until now; cognitive dysfunction-related disorders focus on AD, brain injury, and cognitive disorders after stroke; and the latest research hotspots focus on multidimensional non-pharmacological interventions, including physical exercise and dietary management, and so on.

### 3.6 Author co-citation analysis

**Figure 12:**
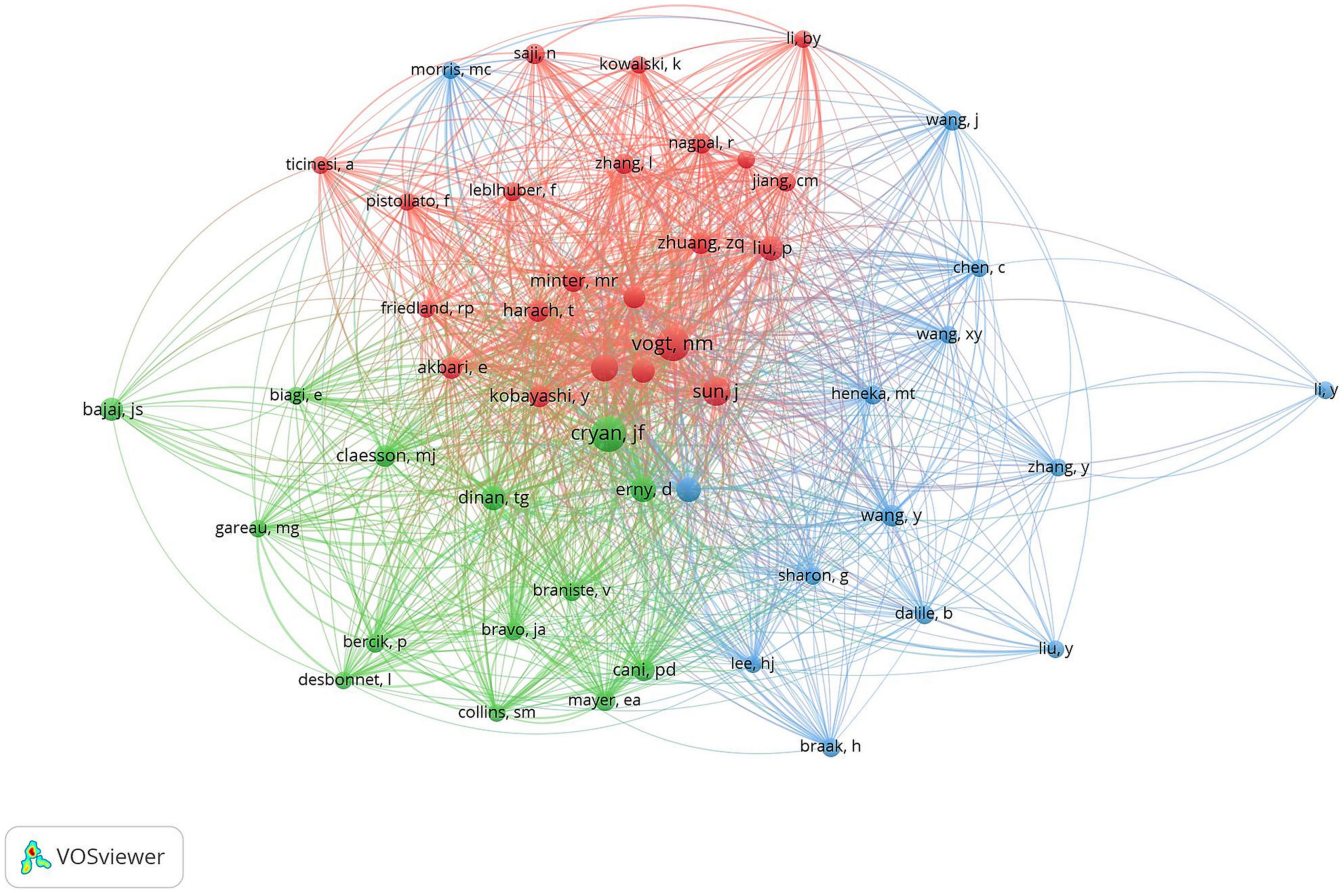
Cluster-based co-author literature collaboration map. The diameter of the circle is positively correlated with the number of publications by the co-cited authors, while the density of the connecting lines intuitively reflects the intensity of collaboration between authors.

VOSviewer was used to construct an author co-occurrence network to identify prolific authors, core collaborative teams, and their academic influence, and Table 6 lists the information of top ten co-cited authors and top ten scholars in centrality ranking. Among them, CRYAN JF topped the list with the highest citation frequency, and his 2019 paper “The Microbiota-Gut-Brain Axis” published in *PHYSIOL REV* ranked second in the literature co-citation analysis. Focusing on the field of gut flora and mental health and neurodegenerative diseases, the team of scholars revealed the molecular mechanisms by which gut flora modulate brain function through vagal pathways, immune signaling molecules, and microbial metabolites. VOGT NM was ranked second in terms of the number of citations for its paper “Gut The VOGT NM team focuses on the association between gut flora and neurodegenerative diseases, and explores the potential link between the gut microbiome and pathological features of AD (Figure 12).

**Table 6:** The top 10 co-cited authors.

Ranking	Counts	Author citation	Centrality	Author citation
1	429	CRYAN JF	0.07	HARACH T
2	408	VOGT NM	0.06	VOGT NM
3	290	CATTANEO A	0.06	BRAVO JA
4	235	ERNY D	0.06	BIAGI E
5	229	SUN J	0.06	BÄCKHED F
6	223	SAMPSON TR	0.05	AKBARI E
7	215	DINAN TG	0.05	CLAESSON MJ
8	208	LIU P	0.05	BENTON D
9	206	HARACH T	0.04	CRYAN JF
10	199	ZHUANG ZQ	0.04	SUN J

### 3.7 Highly cited analysis

**Figure 13:**
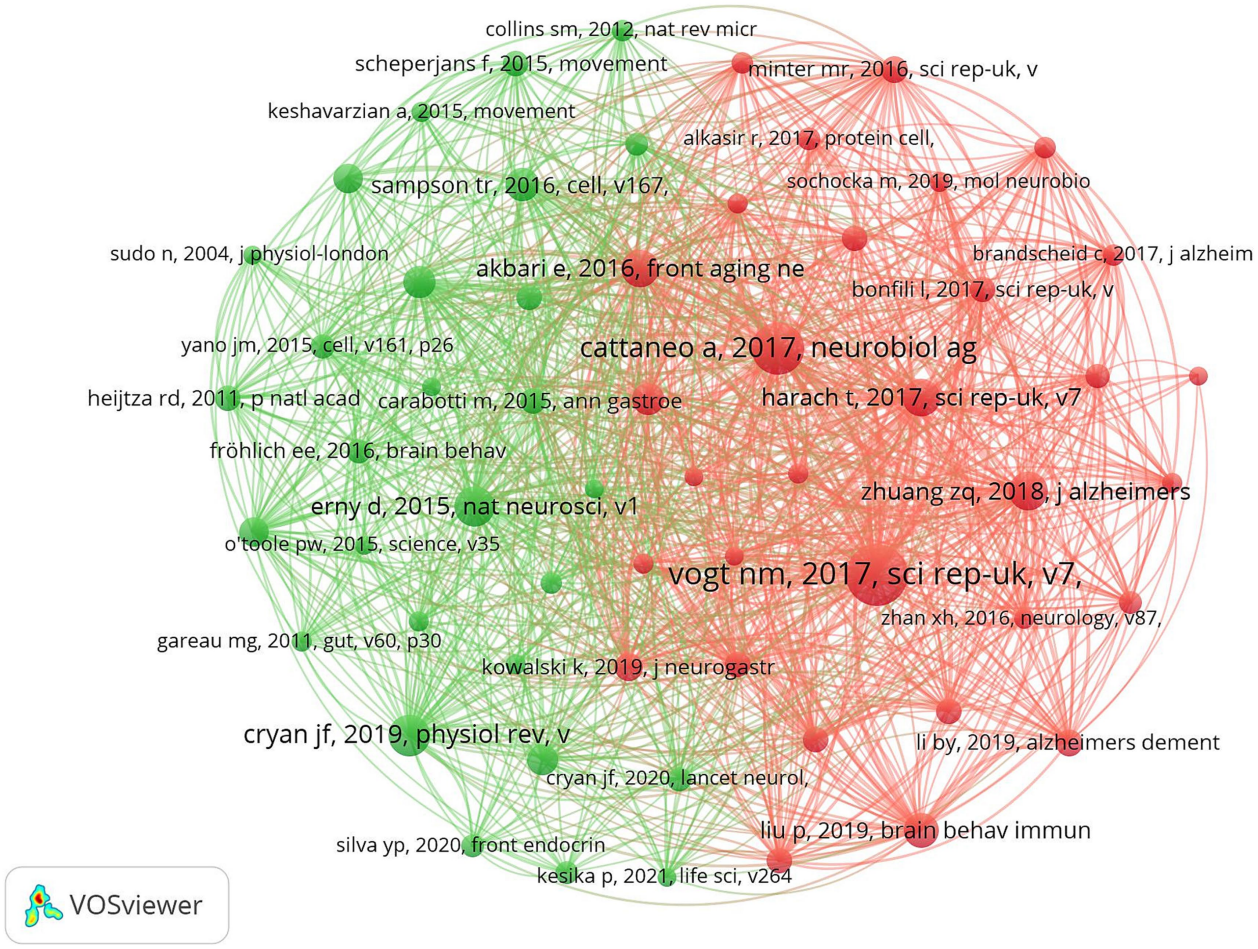
Cluster-based co-citation literature collaboration map. The diameter of the circle is positively correlated with the frequency of the cited literature, while the density of the connecting lines visualizes the correlation between the cited literatures.

Highly cited literature often represents high impact literature in the field. Highly cited literature and high centrality literature focus on the latest research advances within the field. Figure 13 demonstrates the literature co-cited within the field. Table 7 demonstrates the top 10 most frequently cited literature. The article with the highest number of cited literature was “Gut microbiome alterations in Alzheimer’s disease” by Vogt NM published in the *SCI REP-UK* journal, which found a decrease in the thick-walled phylum, an increase in the anaplastic phylum, and a decrease in the bifidobacteria in the microbiome of AD participants. And a correlation was observed between differential abundance genus levels and cerebrospinal fluid biomarkers of AD. A comprehensive analysis of the cited literature shows that the hotspots of current research focus on the clinical efficacy and mechanism of action of intestinal flora to improve cognitive function in patients.

**Table 7:** The top 10 cited literatures.

Ranking	Count	Author	Journal	Title
1	219	Vogt NM, 2017	SCI REP-UK	Gut microbiome alterations in Alzheimer’s disease
2	215	Cryan JF, 2019	PHYSIOL REV	The Microbiota-Gut-Brain Axis
3	191	Cattaneo A, 2017	NEUROBIOL AGING	Association of brain amyloidosis with pro-inflammatory gut bacterial taxa and peripheral inflammation markers in cognitively impaired elderly
4	172	Liu P, 2019	BRAIN BEHAV IMMUN	Altered microbiomes distinguish Alzheimer’s disease from amnestic mild cognitive impairment and health in a Chinese cohort
5	163	Zhuang ZQ, 2018	J ALZHEIMERS DIS	Gut Microbiota is Altered in Patients with Alzheimer’s Disease
6	132	Dalile B, 2019	NAT REV GASTRO HEPAT	The role of short-chain fatty acids in microbiota-gut-brain communication
7	131	Harach T, 2017	SCI REP-UK	Reduction of Abeta amyloid pathology in APPPS1 transgenic mice in the absence of gut microbiota
8	123	Li BY, 2019	ALZHEIMERS DEMENT	Mild cognitive impairment has similar alterations as Alzheimer’s disease in gut microbiota
9	120	Wang XY, 2019	CELL RES	Sodium oligomannate therapeutically remodels gut microbiota and suppresses gut bacterial amino acids-shaped neuroinflammation to inhibit Alzheimer’s disease progression
10	119	Kim MS, 2020	GUT	Transfer of a healthy microbiota reduces amyloid and tau pathology in an Alzheimer’s disease animal model

### 3.8 Journal co-citation

**Figure 14:**
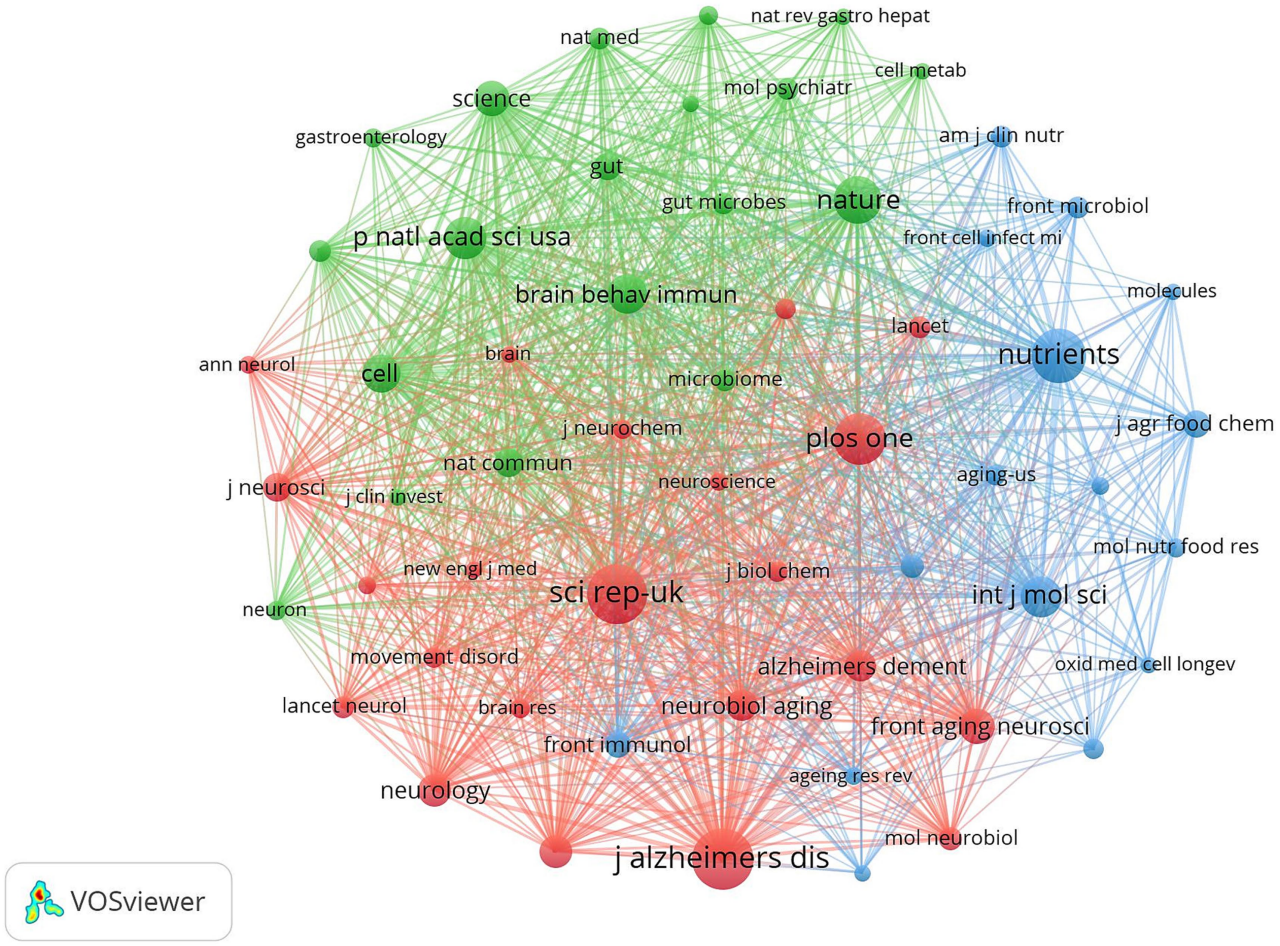
Cluster-based journal co-citation collaboration map. The diameter of the circle is positively correlated with the number of publications in the journal, while the density of the connecting lines visualizes the correlation between journals.

Journal co-citation analysis was performed on the literature data from the Web of Science core ensemble database using cited journals as nodes, and Figure 14 shows the co-citation map of journals in this field. Table 8 shows the top 10 most frequently cited journals in the field. The journal with the highest citation frequency is *SCI REP-UK* (1165). The journal with the highest centrality was *AM J CLIN NUTR* (0.05).

**Table 8:** Top 10 co-cited journals.

Ranking	Counts	Journal	Centrality	Journal
1	1,165	SCI REP-UK	0.05	AM J CLIN NUTR
2	1,091	PLOS ONE	0.03	CELL
3	1,015	J ALZHEIMERS DIS	0.03	INT J MOL SCI
4	971	NATURE	0.03	FRONT AGING NEUROSCI
5	965	NUTRIENTS	0.03	FRONT NEUROSCI-SWITZ
6	884	BRAIN BEHAV IMMUN	0.03	GASTROENTEROLOGY
7	881	P NATL ACAD SCI USA	0.03	ANN NY ACAD SCI
8	852	CELL	0.03	BENEF MICROBES
9	800	INT J MOL SCI	0.03	AM J EPIDEMIOL
10	778	FRONT AGING NEUROSCI	0.02	SCI REP-UK

### 3.9 Journal overlay maps

**Figure 15:**
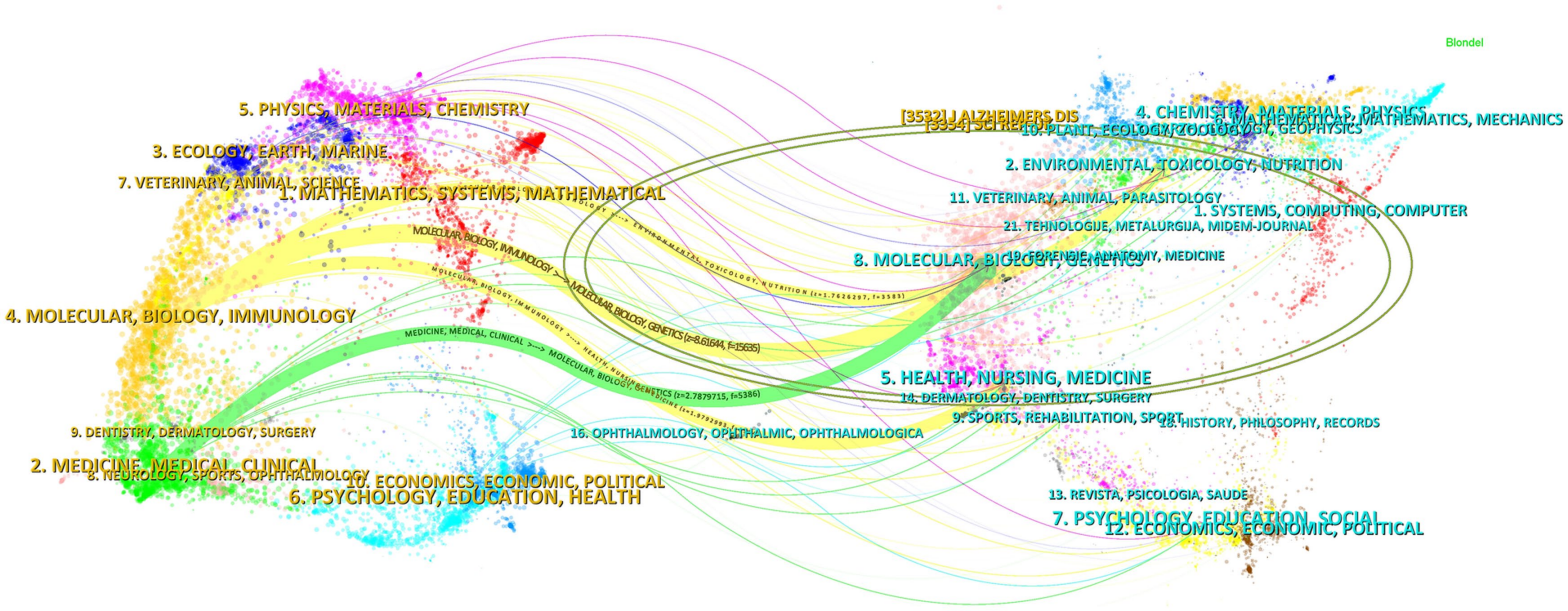
The dual-map overlay of journals. The left side indicates the source journals of the cited literature, the right side indicates the target journals that were cited, and the ellipse represents a subject area or a cluster of journals, with the size of the ellipse reflecting the number or influence of the area/cluster. This figure is automatically generated by CiteSpace software, and journal names are in the form of internationally recognized abbreviations to fit the layout. Full journal names and subject classifications are detailed in Supplementary Table S1.

The dual-map overlay of journals shows the position of a research topic relative to the main research discipline, visualizing the research dynamics of the discipline through the flow of information at the journal level (Chen et al., 2014). As shown in Figure 15, there are four paths in the graph. The yellow path indicates that: journals in the field of MOLECULAR/BIOLOGY/IMMUNOLOGY are usually influenced by MOLECULAR/BIOLOGY/GENETICS, ENVIRONMENTAL/TOXICOLOGY/MUTRITION, and HEALTH/NURSIN/MEDICINE journals. The green path indicates that journals in the field of MEDICINE/MEDICAL/CLINICAL are influenced by MOLECULAR/BIOLOGY/GENETICS.

## 4 Discussion

Based on bibliometric methods, this study used VOSviewer and CiteSpace software to construct a scientific knowledge graph to visualize and analyze the research progress in the field of intestinal flora and cognitive disorders. The study systematically analyzes the research hotspots and evolution paths in this field in terms of publication trends, countries/regions, institutions, journals, authors, and keywords. Unlike traditional single review, this article focuses on four core directions, namely, pathological mechanisms, gut-brain axis regulation, interventions, and gut flora management, and summarizes the mechanism and clinical potential of the emerging fecal microbiota transplantation technology (FMT), as well as provides practical guidance on the management of gut flora in cognitively impaired populations from the perspectives of dietary modification and exercise interventions.

### 4.1 Current status of research

The number of articles published in this field shows a significant upward trend, and the growth rate was be especially obvious after 2021, reaching a historical peak of 404 articles in 2024, which is closely related to the widespread attention to the potential association of intestinal flora in cognitive disorders. With the advancement of biotechnology, more intestinal flora and their mechanism of action will be deeply analyzed, and the number of publications is expected to continue to grow in the future. However, the increase in the number of publications only reflects the increase in research enthusiasm, and cannot directly prove the causal relationship between intestinal flora and cognitive impairment. At the level of research design, animal model studies account for a higher proportion, while the proportion of cohort studies and clinical trials related to human studies has gradually increased in recent years. Human studies have focused more on AD patients, and the overall decrease in gut microbiome species richness in AD patients suggests that gut microbiome studies may facilitate early diagnosis and intervention of AD and other neurodegenerative diseases (Jemimah et al., 2023). However, the limitations of current human studies, such as insufficient sample size and missing longitudinal data, may affect the generalizability of the findings. It is worth noting that gender differences have received extensive attention from researchers – preliminary evidence suggests that the abundance of Lactobacillus spp. in the gut flora of females may be affected by estrogen levels and associated with cognitive decline (Peters et al., 2022), and this hypothesis needs to be verified by large-scale gender-balanced studies in the future.

In terms of the number of articles from countries/regions and institutions, the country with the largest number of articles is China (798 articles), followed by the United States (287 articles) and Italy (132 articles). In terms of centrality, Australia (0.81), Saudi Arabia (0.71), and the Netherlands (0.47) have high centrality, indicating close cooperation with other countries/regions. China had a high research heat in this field and has achieved certain research results in this field, but the centrality was low, and the communication with other countries/regions is low, so it needs to strengthen the communication and cooperation between countries/regions in the future, to further validate the correlation between gut flora and cognitive impairment. From the institutional co-occurrence network map and hotspot map, it can be seen that Huazhong University of Science & Technology (41 articles) is the institution with the largest number of articles and higher influence in this field. This is followed by Capital Medical University and Harvard University. Harvard Medical School (0.47), Boston University (0.44), and Zhejiang University (0.3) are the top three institutions in terms of centrality. Sex ranked top three institutions. Among the institutions, the number of articles published by Chinese institutions is high, and the centrality of Zhejiang University is the highest among Chinese institutions, which indicates that Zhejiang University is actively engaged in exchanges and cooperation with other institutions to promote the continuous development of this field.

The most cited journal is SCI REP-UK (1,165 articles), which publishes original research from all fields of natural sciences, psychology, medicine, and engineering. Covering all areas of the natural sciences, psychology, medicine, and engineering, this journal has more publications in the area of intestinal flora in cognitive disorders, with the highest citation frequency and high impact. The journal with the highest centrality is AM J CLIN NUTR (0.05), which is the highest rated peer-reviewed primary research journal in the field of nutrition and dietetics, focusing on original research related to human and clinical nutrition, as well as public health and epidemiologic research related to human nutrition.

### 4.2 Research hotspots and trends

Keyword analysis can intuitively and comprehensively reveal the research hotspots and trends in the field and provide directions for subsequent research (Li et al., 2022). Comprehensive keyword and literature co-citation analyses revealed that current research in the field of intestinal flora and cognitive disorders focuses on the following directions: at the disease level AD is the core research object; at the mechanism level oxidative stress and inflammation have long been focused on as the key pathological mechanisms; at the pathway level the gut-brain axis has been identified as an important signaling pathway in which the intestinal flora affects the cognitive function; and at the metabolite level the SCFAs are the most widely studied microbial metabolites. In addition FMT has attracted much attention in recent years as an emerging intervention to regulate intestinal flora and dietary management and exercise have become the latest research hotspots in the field of intestinal flora management. It is worth noting that most of the existing studies are based on observational data or animal models and their conclusions mainly reflect the correlation between intestinal flora and cognitive impairment but no direct causal link has been established.

#### 4.2.1 Correlation analysis of oxidative stress and inflammatory response

The results of the keyword analysis show that oxidative stress and inflammatory responses in the gut flora maintain a high level of research fervor. Relevant studies have shown that there is a significant correlation between gut flora imbalance and neuroinflammation and cognitive decline, mainly in terms of exacerbation of oxidative stress, mediation of inflammatory signaling through the gut-brain axis, and modulation of metabolites. Neuroinflammation and metabolic abnormalities associated with oxidative stress occurring in the brain can have an impact on cognitive function (Song et al., 2021). When oxidative stress occurs in cells, redox signaling pathways are disrupted in the cell, and the generation of reactive oxygen species levels exceeds the increase in antioxidant levels, and the imbalance between the two affects the organism and is a major cause of many neurological disorders (Sun et al., 2020). Oxidative stress led to cellular damage by mediating three major reactions: lipid peroxidation, protein oxidation and nucleic acid damage (Singh et al., 2019). Reactive oxygen species act as secondary messengers in various parts of the brain that maintain synaptic plasticity (Massaad and Klann, 2011) and play an important role in cognitive function. Aging is closely related to oxidative stress, and an imbalance between cellular defense and antioxidant mechanisms can lead to reactive oxygen species destroying the organism’s antioxidant defense system (Li et al., 2018). Oxidative stress produces highly active oxidizing factors that lead to intracellular DNA damage, protein denaturation, and lipid peroxidation (Dong et al., 2023), causing damage to biomolecules and cell membranes in the human body and leading to a decline in cellular function (He et al., 2019). In AD, oxidative stress promotes A*β* deposition and tau hyperphosphorylation, leading to loss of synapses and neurons (Forman and Zhang, 2021). An 8-week red yeast rice dietary intervention in mice was found to reduce oxidative stress and NF-κb-mediated inflammatory responses, which significantly improved cognitive performance and reduced oxidative stress-related damage (Huang et al., 2022). In daily life, human consumption of selenium- and/or zinc-enriched eggs (SZE) may improve cognitive function by attenuating oxidative stress and inflammatory responses as well as maintaining healthy gut flora (Liu et al., 2024).

The organism exhibits a chronic progressive pro-inflammatory response during aging, and the inflammatory response gradually destroys the balance of the intestinal flora, severely affecting the composition of the intestinal flora and leading to a gradual decline in the diversity and stability of the microbiota (Hu et al., 2016). And the imbalance of intestinal flora such as the decrease of Bacteroides phylum and the increase of Thick-walled phylum can lead to the increase of intestinal epithelial barrier permeability and the release of pro-inflammatory cytokines into the bloodstream, such as IL-6 and TNF-*α*, which can activate microglia through the circulatory system to promote neuroinflammatory response (Medzhitov, 2007), and then damage neurons and synaptic function. In AD patients, extracellular amyloid-β plaques and intracellular tau protein accumulation activate microglia, triggering chronic inflammation and neuronal death (Sato et al., 2018), which results in cognitive impairment. Chronic inflammation underlies the association between gut microbial characteristics and cognitive impairment (Liang et al., 2022). Abnormally functioning microglia can initiate signaling cascades that lead to neuroinflammation (Carlessi et al., 2021). SCFAs produced by the metabolism of intestinal flora can cross the blood–brain barrier and inhibit neuroinflammatory responses, and SCFAs can limit the production of pro-inflammatory cytokines, such as IL-1 (Perrone et al., 2020), reduce blood–brain barrier permeability, and exert anti-neuroinflammatory effects (Varela et al., 2015). Histamine produced by gut microbes exerts immunomodulatory and anti-inflammatory properties (Pugin et al., 2017). In daily diet, apple polysaccharides extracted from apple fruits can enhance antioxidant capacity, inhibit the activation of NF-κB signaling pathway, exert anti-inflammatory and antioxidant functions, and repair the intestinal mucosal barrier (Zhang et al., 2024). Researchers have found that probiotics may improve cognitive performance in patients with AD or MCI by reducing levels of inflammatory and oxidative biomarkers (Den et al., 2020).

Microbiota dysbiosis is not only a driver of localized intestinal inflammation, but also creates a “bidirectional vicious circle” with central neuroinflammation through a systemic inflammatory response. Dysbiosis of intestinal flora disrupts the integrity of the intestinal barrier, leading to the entry of lipopolysaccharides (LPS) and other pathogen-associated molecular patterns into the circulatory system, activation of peripheral immune cells, and infiltration of proinflammatory factors (IL-6, TNF-*α*) through the blood–brain barrier into the CNS, activating microglial cells and triggering neuroinflammation (Sorrentino et al., 2017). cells and trigger neuroinflammation (Cryan et al., 2020). Neuroinflammation further exacerbates dysbiosis by altering intestinal motility, mucus secretion, and the immune environment, and in patients with AD, reduced diversity of intestinal flora is highly correlated with Aβ deposition and tau protein phosphorylation in the brain (Hung et al., 2022). Animal model studies have shown that mice transplanted with fecal flora from AD patients exhibit increased intestinal barrier permeability, elevated peripheral inflammatory markers, and concomitant hippocampal neuroinflammation and cognitive deficits (Zhang D. et al., 2023). This process involves mechanisms of immune-metabolic axis imbalance, mitochondrial dysfunction and blood–brain barrier disruption. When the immune-metabolic axis is imbalanced, dysregulated flora affects the differentiation of peripheral regulatory T cells (Treg) by altering bile acid metabolism and tryptophan catabolic pathways, weakening immune tolerance, and promoting CNS neuroinflammation (Campbell et al., 2020); mitochondrial dysfunction leads to the fact that metabolites of intestinal flora can directly impair neuronal mitochondrial function and increase reactive oxygen species generation, which in turn accelerates neuronal inflammation. Synergize with neuroinflammation to accelerate neuronal apoptosis (Kalyanaraman et al., 2024); after disruption of the blood–brain barrier, pro-inflammatory factors up-regulate matrix metalloproteinases in chronic inflammatory states, degrade blood–brain barrier tight junction proteins, and promote the penetration of peripheral inflammatory factors and neurotoxic substances into the brain parenchyma (Kurz et al., 2022). Taken together, microbiota dysbiosis is a central pathologic link in the progression of cognitive impairment through multidimensional modulation of the neuroinflammatory framework. Interventions targeting the gut flora may provide new strategies for the treatment of neuroinflammatory diseases by restoring flora homeostasis and inhibiting inflammatory pathways.

#### 4.2.2 Correlation between the mechanism of action of the gut-brain axis and cognitive impairment

From the keyword analysis, it can be seen that the gut-brain axis plays an important role in the study of gut flora and cognitive impairment. The enteric nervous system is a key link in communication between the gastrointestinal tract and the central nervous system (Rao and Gershon, 2018). Playing a role in the regulation of the body by gut flora through endocrine, humoral, metabolic, and immune-mediated signaling pathways, the entero-brain axis mainly consists of the autonomic nervous system, the hypothalamic–pituitary–adrenal axis, and neural networks within the gastrointestinal tract (Toader et al., 2024). Intestinal flora affects the function of the gut-brain axis by influencing alterations in the secretion and release of bioactive peptides from enteroendocrine cells (Barton et al., 2023). Moreover, intestinal neurons can communicate with each other and the central nervous system through neural, endocrine and immune pathways to maintain gut health (Morais et al., 2021). The direct effect of gut flora on neuromitochondria also affects the function of the gut-brain axis (Zhu et al., 2022); SCFAs are able to cross the blood–brain barrier and affect mitochondria in the brain to regulate energy production, and GABA, 5-hydroxytryptophan, and dopamine can also affect mitochondrial function (Bicknell et al., 2023), which can affect cognitive function. The gut-brain axis has been recognized as a potential target for treating neurodegenerative diseases and improving cognitive function (Qu et al., 2024). Vagus nerve stimulation has been found to have an anti-inflammatory effect on the gut microbiota (Wang and Wang, 2016). Signals from the gut and bacteria have an impact on brain function (Lynch and Pedersen, 2016), and Aβ or Tau proteogen fibers injected into the colon or brain lysates from AD patients propagate from the gut to the brain via the vagus nerve, where inflammation activates C/EBPβ/*δ*-secretase and triggers AD-related pathological changes and cognitive dysfunction in the gut (Chen et al., 2021). Neuromodulation plays a significant role in modulating the function of the brain-gut axis, such as deep brain stimulation (DBS), vagus nerve stimulation (VNS), spinal cord stimulation (SCS), and transcranial magnetic stimulation (TMS), which modulate the function of nerves through stimulation of the corresponding brain regions and influence the cognitive function of the brain through the gut microbiota-gut-brain axis (Aljeradat et al., 2024).

#### 4.2.3 Potential role of short-chain fatty acids in improving cognitive functions

According to the results of keyword analysis and literature co-citation analysis, SCFAs have become a hot research topic in this field. The interaction between the microbiota and the immune system is mainly mediated by microbe-derived metabolites, such as SCFAs and tryptophan metabolites (Levy et al., 2016). The production of SCFAs through soluble fiber fermentation is one of the major ways in which the microbiota promotes gut health. SCFAs, as key metabolites of gut microbes, are involved in the regulation of cognitive functions through various pathways, including anti-inflammation, modulation of the gut-brain axis, and influence on neurotransmitters and synaptic plasticity. Butyrate is considered to be the most important SCFAs and consists of the phylum Thick-walled Bacteria, Anaplasma species and Bifidobacterium species (Nandwana et al., 2022). Odorobacterium, Aeromonas butyricum and Bacteroidetes, are the bacteria that produce SCFAs with significant anti-inflammatory and immunomodulatory effects (Cryan et al., 2020). The intestinal flora acts by modulating vagal activity and secreting neurotransmitters, mainly *γ*-aminobutyric acid (GABA), serotonin, and SCFAs (Silva et al., 2020). SCFAs are produced in the cecum and colon by anaerobic fermentation of small organic molecules, mainly non-digestible dietary carbohydrates, and these compounds are not only cross-fed with other bacteria, but also efficiently absorbed by the large intestine (Zhang Y. et al., 2023). SCFAs play a key role in digestion, immunity and central nervous system function (van de Wouw et al., 2018). SCFAs are absorbed by intestinal epithelial cells, participate in host metabolism, and can cross the blood–brain barrier via monocarboxylic acid transporters (Mitchell et al., 2011), thus improving nutrition of brain regions associated with cognitive function and cognitive function. A variety of intestinal flora play an important role in the fermentation of SCFAs (Xia et al., 2021), and adequate SCFAs regulate proteins in the tight junctions between cells, thus maintaining the barrier between colon contents and body tissues against bacteria and their toxic metabolites (Zhu et al., 2021). The pathways of action of SCFAs through molecular mechanisms can improve cognitive impairment, modulate AD-related marker deposition, modulate blood–brain barrier permeability as well as provide anti-inflammatory and anti-apoptotic effects (Qu et al., 2024). SCFAs can also affect brain function through inhibition of his – tone deacetylase and induction of enteroendocrine signaling (Cryan et al., 2019). Studies have shown that SCFAs can enhance the expression of proteins associated with synaptic plasticity (Tan et al., 2014) and can act as a broad-spectrum histone deacetylase (HDAC) inhibitor, which can be utilized to modulate HDAC in AD, enhance gene expression, attenuate pathological changes in patients with AD, and improve cognitive function (Govindarajan et al., 2011). Lee H et al. in a study, found that butyrate increased the expression of neurotrophic factors, and butyrate attenuated radiation-induced cognitive deficits by attenuating the inhibition of hippocampal cAMP-responsive element-binding protein phosphorylation or by promoting the expression of brain-derived neurotrophic factors (Lee et al., 2019). Shi L et al. found that in microglia, butyrate attenuated radiation-induced cognitive deficits through the up-regulation of phosphoinositide 3-kinase (PI3K)/protein kinase B (AKT)/cAMP-responsive element binding (CREB)/BDNF signaling pathway (Shi and Tu, 2015), which promotes long-term enhancement and synaptic plasticity, thereby improving memory function in patients. Prebiotic mannan oligosaccharide (MOS) remodels the gut microbiota and promotes the formation of neuroprotective metabolites SCFAs, and an 8-week MOS treatment significantly improved cognitive function and spatial memory, significantly reduced A*β* accumulation in the cerebral cortex, hippocampus, and amygdala in a 5xFAD transgenic mouse model of AD, and MOS treatment significantly balanced the cerebral redox state and suppressed the Neuroinflammatory responses (Liu et al., 2021). Li JM et al. found that intestinal dysbiosis was a key factor in hippocampal neuroinflammation induced by high fructose diet in C57BL/6 N mice, and SCFAs could stimulate NLRP6 inflammatory vesicles and ameliorate the damage to the intestinal epithelial barrier, which prevented hippocampal neuroinflammation and neuron loss induced by high fructose diet (Li et al., 2019).

#### 4.2.4 Evidence for the relevance of fecal microbiota transplantation techniques to improve cognitive functioning

Based on the results of keyword clustering, it can be seen that FMT has become a hot topic and trend of research in recent years. FMT is a method of altering the microbial composition of the intestinal tract. FMT is a therapeutic intervention that transfers donor feces into the recipient’s gastrointestinal tract. This procedure directly modifies microbial composition and improves functional status (Smits et al., 2013). FMT was first clinically applied in 1958 for the treatment of pseudomembranous small bowel colitis (Xiao et al., 2022), Sun, J et al. showed that FMT treatment ameliorated cognitive deficits and reduced brain deposition of amyloid *β* in APPswe/PS1dE9 transgenic mice, and that tau protein phosphorylation, as well as reduced levels of Aβ40 and Aβ42, and increased synaptic plasticity were experimentally observed in mice (Sun et al., 2019). Kim M-S et al. found that transplantation of feces from healthy mice transplantation of targeted microbiome ameliorated plaque deposition and neuroprogenitor fiber tangles, immune dysfunction, and memory deficits (Kim et al., 2020). An animal experiment conducted by M.-F. Sun et al. gavaged mptp-induced Parkinson’s disease mice with 200 μL of cecal bacterial suspension in healthy mice for 7 days, which, by decreasing the TLR4/TBK1/NF-jB/TNFa signaling pathway in both the intestine and brain activity and reduced brain glial cell activation (Sun et al., 2018). Another study also demonstrated improved cognitive performance and reduced amyloid accumulation and improved synaptic plasticity in an AD mouse model after FMT (Sun et al., 2019). FMT can improve cognitive function by decreasing the accumulation of amyloid b and upregulating the expression of synaptic markers (PSD-95, synaptophysin-1) in Alzheimer’s disease (Hediyal et al., 2024). FMT improves cognitive function by increasing the expression of synaptic markers (PSD-95, synapsin-1) and decreasing Ab accumulation and neuroinflammatory markers (COX-2, CD11b) to improve cognition (Sun et al., 2019). FMT restored the levels of *Bifidobacterium bifidum* and *Bifidobacterium bifidum* in 3xTg-AD mice, which significantly delayed the progression of AD (Bonfili et al., 2018). A 5XFAD mouse model showed significant improvements in new object recognition and spatial recognition in wild-type donor mice treated with FMT for 7 days resulted in significant improvement in new object recognition and spatial memory, accompanied by a reduction in amyloid pathology (Elangovan et al., 2022). After FMT intervention, there was a significant improvement in gut microbial *α*-diversity and β-diversity indices in mice (Zhan et al., 2018). Kim M-S et al. found that FMT ameliorated Aβ deposition, neurogenic fibrillar tangles, glial cell reactivity, and cognitive deficits in the brains of AD mice through their study (Kim et al., 2020). During the clinical application of FMT, healthy donors are required to pass multiple screenings to exclude infectious diseases, metabolic diseases and history of psychiatric disorders. Fecal samples should be tested for pathogenic microorganisms and SCFAs to ensure microbial diversity (Shannon index >3.5) (Cammarota et al., 2017). Transplantation methods mainly include oral capsule and colonoscopic infusion, with oral capsule being lyophilized fecal flora encapsulated in an enteric-coated capsule, and colonoscopic infusion of 200–300 mL of the flora suspension injected directly into the colon through a colonoscope. Regular testing of intestinal flora composition (16S rRNA sequencing) and plasma inflammatory markers (e.g., IL-6, CRP) was required after treatment, and the long-term safety and standardized preparation process of FMT still need to be further validated, and ethical review needs to focus on donor privacy and subjects’ right to know (Sandhu and Chopra, 2021). Currently, there is limited evidence of RCT in humans, and the long-term safety and individual differences in colony transplantation remain significant challenges.

#### 4.2.5 Moderating effects of dietary management and exercise on cognitive functioning

The regulation of gut microbiota composition and function and its impact on brain and behavior is dependent on the influence of diet (Schneider et al., 2024). Studies have shown that different dietary patterns determine different patterns of gut microbiota composition, with individuals adhering to animal-based diets exhibiting significant compositional and functional characteristics compared to those adhering to vegetarian diets (Djekic et al., 2020). The Western Diet (WD) and Mediterranean Diet (MD) are the main diets that have been studied for their effects on the gut microbiota and subsequent health outcomes. The WD is high in simple sugars, saturated fats, and salt and low in fiber, complex carbohydrates, and micronutrients (Kendig et al., 2021). Ultra-processed foods which are usually high in sugar, salt and fat, characterize WD and are thought to be a predisposing factor for low-grade systemic inflammation and oxidative changes, which contribute to the onset and progression of neurodegenerative disorders (Martínez Leo and Segura Campos, 2020), and high-fat diets can have an impact on affecting the intestinal flora, which can have an effect on the proper functioning of the brain (Devkota et al., 2012). Increased levels of *Bacillus thuringiensis*, Bacillus anthropophilus, Escherichia, Shigella and Klebsiella are associated with the intake of high-fat, high-sugar and processed foods and can lead to decreased levels of Lactobacillus, Roseobacter and Bacillus anthropophilus (Dissanayaka et al., 2024). Short-term intake of a high-fat diet leads to hippocampus-dependent spatial memory impairment in rats (Khazen et al., 2019). WD disrupts the balance of the intestinal flora, leading to a compromised intestinal barrier and an increase in permeability (Zhang et al., 2019). WD intake will lead to systemic inflammation and cognitive dysfunction (Jena et al., 2022). The Mediterranean diet (MD) was associated with characteristic alterations in the gut microbiota mediated through dietary fiber, extra virgin olive oil, and polyunsaturated fatty acids (Barber et al., 2023). The MD is rich in fermentable dietary fiber and is associated with an increased abundance of fiber-fermenting bacteria leading to an increase in short-chain fatty acids in the gut and blood (Wu et al., 2011). Omega-3 fatty acids, antioxidants, and polyphenols in the MD diet can inhibit the AD-related neuroinflammation (McGrattan et al., 2019). A modified Mediterranean ketogenic diet modulates the gut microbiome and short-chain fatty acids and improves mild cognitive impairment (Nagpal et al., 2019). MD not only maintains the normal function of the blood–brain barrier (Braniste et al., 2014), but also improves gut microbial composition and diversity (De Filippis et al., 2016). Long-term adherence to the Mediterranean diet has been associated with elevated levels of intestinal SCFAs and reduced risk of AD (Ballarini et al., 2021). Dietary fiber intake is 25–30 g per day, with preference given to whole grains, legumes, fruits and vegetables. Prebiotics (e.g., oligofructose, inulin) may increase the production of SCFAs by promoting the proliferation of bifidobacteria and lactobacilli (Holscher, 2017). The following principles need to be followed to implement MD: fat source of extra virgin olive oil as the main fat source (30–50 mL per day), reducing the intake of saturated fats (e.g., red meat, butter); protein intake needs to be at least 3 times per week of fish (especially salmon and sardines, which are rich in omega-3 fatty acids), and moderate intake of poultry and beans; and daily intake of polyphenol-rich foods (e.g., dark berries, dark chocolate, green tea) to suppress neuroinflammatory responses (Vauzour et al., 2017).

Exercise can be used as an external stimulus to influence the gut and its associated gut microbiota. Exercise modulates the production of gut microbial metabolites such as SCFAs and endogenous endotoxins, which play an important role in regulating the function of the gut flora (Herrera-Rincon et al., 2020). Sustained exercise alters the composition of the gut flora and modulates neurogenesis and neuroinflammation may be the main mechanism by which exercise-induced gut flora improves cognitive function (Ogbonnaya et al., 2015). Peng M et al. demonstrated that high-intensity interval training (HIIT) and moderate-light training (MICT) modulate the composition of the gut microbiota and its metabolite lipopolysaccharides by modulating gut microbiota composition and their metabolite lipopolysaccharides through gut microbiota-gut-brain axis affecting cognitive functions in the brain (Peng et al., 2024). A study by Wang G et al. found that HIIT altered microbiota composition and diversity in mice and dynamically altered the gut microbiota profile in middle-aged mice (Wang et al., 2020), leading to improved cognitive functions. HIIT is recommended for 20–30 min three times per week and consists of four sets of 4-min high-intensity exercise (e.g., cycling, brisk running, up to 85–95% of maximal heart rate) with a 3-min interval of low-intensity recovery (Blackmore et al., 2024); moderate-intensity continuous training is recommended for 45–60 min of moderate-intensity exercise five times per week (e.g., brisk walking, swimming, up to 60–70% of maximal heart rate) (Yu et al., 2022). Exercise promoted the production of SCFAs by increasing intestinal blood flow and mechanical stimulation. A study in older adults found that 12 weeks of MICT significantly increased the abundance of Mycobacterium anomalum phylum, decreased the thick-walled Mycobacterium anomalum phylum/ Mycobacterium anomalum phylum ratio, and was positively correlated with improved cognitive function (Mailing et al., 2019).

#### 4.2.6 Potential impact of gender differences

Differences in gut flora diversity between genders have been associated with the risk of cognitive impairment, with a more pronounced decline in female cognitively impaired patients and correlation with Aβ protein deposition, and in males more often associated with elevated inflammatory factors (Cuervo-Zanatta et al., 2021). Estrogen is neuroprotective in women and may delay cognitive impairment by modulating gut flora composition, increasing short-chain fatty acid-producing flora, and reducing neuroinflammation (Baker et al., 2017). Women have higher levels of SCFAs produced by gut flora, which may reduce neurotoxin infiltration by enhancing blood–brain barrier integrity, while men have higher levels of trimethylamine oxide (TMAO) produced by flora metabolism, which may exacerbate cerebrovascular injury and cognitive decline (Sullivan et al., 2025). Gender differences in immune responses mediate gut-brain axis regulation. Females exhibit stronger immune reactions to gut microbiota disruption, which may lead to more pronounced neuroinflammatory and cognitive symptoms such as anxiety and memory loss, while males are more susceptible to systemic inflammation triggered by flora dysregulation due to lower activity of immune-suppressing cells (e.g., Treg cells) (Rizzetto et al., 2018). Gender-specific effects occur with dietary and probiotic interventions, with women responding more sensitively to dietary fiber and probiotics, potentially improving cognition through modulation of the flora-estrogen axis (Honda et al., 2024); men require higher doses of prebiotics or targeting of specific metabolic pathways (e.g., tryptophan metabolism) to achieve a similar effect (Roth et al., 2021).

## 5 Limitations

First, this study included research data from the Web of Science Core Collection database, which, despite its authority in bibliometric analysis and support for a standardized search process, did not include other databases such as Scopus or PubMed or literature in other languages, which may limit the breadth of literature coverage. The reflected research content and hotspots may not cover all the content in this field, and there may be a certain bias; secondly, there is no quality evaluation of the included literature, and the quality of the included literature may be uneven, which will have a certain impact on the analysis results. Future studies may further optimize the scope of analysis through multi-platform data integration to improve the comprehensiveness of the data, and quality evaluation of the included literature may be performed to improve the quality of the included articles.

## 6 Conclusion

The article reviews summarize the history and current status of research in the field of intestinal flora in cognitive disorders from 2012–2025. The research trend showed that the number of publications in the field had increased dramatically in recent years, indicating that the field is currently receiving a high level of attention. China became the country with more publications, and Huazhong University of Science & Technology and Kim, Dong-Hyun were the organizations and individuals with more publications. The journal with the highest citation frequency was *SCI REP-UK*. Oxidative stress and inflammatory response became the main research hotspots for gut flora to improve cognitive impairment in patients. The gut-brain axis establishes a link between the brain and the gut, which plays an important role in the study of the mechanism of action of gut flora to improve cognitive dysfunction. SCFAs, as a focus of research on the metabolism of intestinal microorganisms, play an obvious role in reducing inflammation and oxidative stress, and have a significant effect on the improvement of cognitive function. FMT technology, as an emerging approach applied to gut flora, plays an active role in improving cognitive function. Future studies may focus more on gender differences in the role of gut flora in the modulation of cognitive function. Gut flora can be regulated by factors such as diet and exercise. Changing the intestinal flora through lifestyle modification may be an effective strategy to prevent and improve cognitive dysfunction in the future. The current study is more reflective of correlation, and future experiments are needed to verify causality.
